# Efficient QM/MM Modeling of Enzymatic Reactions Combining PathCV with OPES

**DOI:** 10.1021/acs.jctc.6c00492

**Published:** 2026-05-27

**Authors:** José Pablo Rivas-Fernández, Martin Calvelo, Mert Sagiroglugil, Qinghua Liao, Carme Rovira

**Affiliations:** † Departament de Química Inorgànica i Orgànica & IQTCUB, 16724 Universitat de Barcelona, Martí i Franquès 1, 08028 Barcelona, Spain; ‡ Institució Catalana de Recerca i Estudis Avançats (ICREA), Passeig Lluís Companys, 23, 08020 Barcelona, Spain

## Abstract

Hybrid quantum mechanics/molecular mechanics (QM/MM) simulations combined with enhanced sampling are powerful tools for studying enzymatic reaction mechanisms, but their application remains limited by the short time scales accessible at the QM/MM level and by the difficulty of defining efficient collective variables. Here, we present a practical protocol that combines a path collective variable (PathCV) with the exploratory variant of on-the-fly probability-enhanced sampling (OPES_E_) to accelerate reactive transitions and reconstruct free energy profiles in enzymatic QM/MM simulations. A PathCV is constructed from a preliminary reactive trajectory and then used as a reaction coordinate in a subsequent biased simulation, avoiding costly on-the-fly path refinement. We also introduce a block-selection strategy that isolates statistically reliable trajectory segments to enable robust free energy reconstruction from time-dependent biased trajectories. A systematic analysis of OPES_E_ parameters further identifies practical deposition and adaptive sigma stride settings. We apply this protocol on three mechanistically distinct enzymes: polysaccharide lyase *Ps*Alg7A, human *O*-GlcNAcase, and SARS-CoV-2 main protease. In all cases, PathCV-guided OPES_E_ promotes multiple reactive transitions within QM/MM-accessible simulation times and outperforms alternative biasing strategies (OPES, metadynamics, and well-tempered metadynamics) in terms of sampling efficiency. Overall, our work provides a practical guide for computationally demending enhanced sampling QM/MM studies of enzymatic reactions.

## Graphical abstract

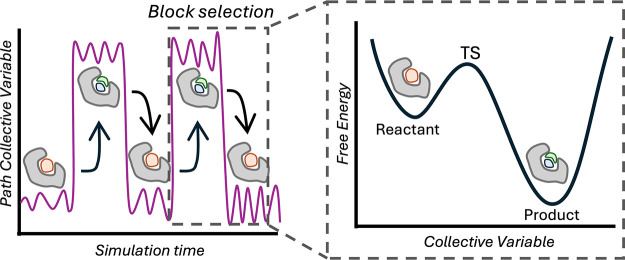

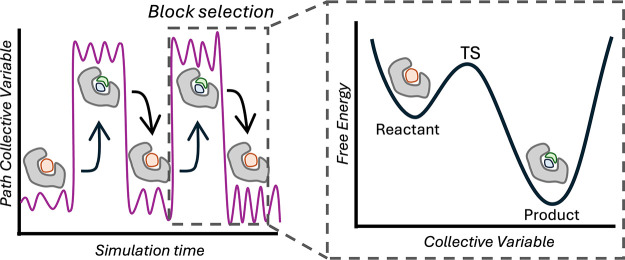

## 1 Introduction

In recent years, advances in computational power and algorithm development have significantly expanded the capabilities of molecular dynamics (MD) simulations, enabling the study of increasingly complex systems across disciplines.^1–3^ Yet, the accurate description of long-time scale rare events—such as transitions between metastable states separated by substantial kinetic barriers—remains a central challenge. To overcome this, MD simulations are frequently combined with enhanced sampling techniques designed to accelerate rare events and facilitate exploration of the underlying free energy landscape (FEL).^4^

Many enhanced sampling approaches rely on applying an external bias along carefully chosen collective variables (CVs) that capture the slow degrees of freedom relevant to the process of interest. Representative examples include umbrella sampling (US),^5,6^ adaptive biasing force (ABF),^7^ and metadynamics (MetaD).^8^ In MetaD, Gaussian-shaped bias potentials (“hills”) are deposited along selected CVs, gradually filling free energy wells and allowing escape from local minima. The accumulated bias then provides an approximate estimate of the FEL.^9^ In the context of enzymatic catalysis, these techniques are commonly combined with hybrid quantum mechanics/molecular mechanics (QM/MM) simulations, in which the chemically active region is treated with quantum mechanics methods, while the surrounding environment is described with classical force fields.^10,11^ This multiscale strategy has successfully revealed mechanistic insights into a broad range of enzymes, from the first work on Hsc70 ATPase by Boero et al.,^12^ to ribozymes,^13,14^ decarboxylases,^15^ endonucleases,^16^ and carbohydrate-active enzymes,^17,18^ among others.^19^ These works have identified previously unknown sugar conformations useful for activity-based probe design^20^ and catalytic motifs amenable to mutagenesis, enabling engineering of enzymes with new or promiscuous activities.^21,22^

Despite their utility, QM/MM MetaD simulations for enzymatic reactions remain computationally demanding, and a sufficient number of reactive transitions to ensure proper sampling is often not achieved. A pragmatic and widely adopted compromise is to require a single recrossing over the transition state (TS), a strategy validated in early benchmarks^23^ and more recent applications.^19,24–26^ Although this approach does not allow estimation of a statistical error, it provides a balance between computational feasibility and mechanistic insight while limiting exploration of spurious pathways that may arise from the use of suboptimal CVs. Nonetheless, the selection of CVs remains a critical challenge: the small number of CVs that can be biased in QM/MM simulations (typically one to three, due to the high computational cost) can make it difficult to capture all slow degrees of freedom governing the reaction with sufficient accuracy.^4^ Furthermore, when CVs are defined as combinations of structural descriptors, such as distances or angles, correlations between them do not alter the underlying FEL but can distort its projection onto the chosen CV space, i.e., the computed FEL. In these cases, a nonunique or degenerate description of the reaction coordinate is obtained,^9^ which fails to capture the nonlinear and often asynchronous of the reactive process. Because enhanced sampling methods deposit bias only along these CVs, an inadequate definition can lead to inaccurate barriers and hinder transitions between metastable states.

A more robust alternative is provided by path-like collective variables (PathCVs), which encode progress along a reference reaction pathway and integrates multiple descriptors into a single, chemically interpretable coordinate. Several formulations have been proposed, including the string method,^27^ the path-CV of Branduardi et al.,^28^ and the alternative formulation introduced by Díaz-Leines and Ensing.^29,30^ In the latter, the PathCV comprises a progress parameter ***s*** (ranging from 0 to 1) and a distance parameter ***z*** describing orthogonal deviation from the path. This path is discretized into nodes in the descriptor space and, as the simulation proceeds, each node is iteratively displaced toward the maximum of the sampled distribution in the hyperplane perpendicular to the path (see Methods for more details). This data-driven updating procedure ensures that the PathCV contains the dominant transition path and provides a physically grounded reaction coordinate for complex systems with many degrees of freedom.^31^

Recently, a MetaD-inspired algorithm, On-the-fly Probability Enhanced Sampling (OPES), was introduced to accelerate convergence in the FEL estimation.^32^ OPES builds the bias potential on-the-fly (see Methods) from the reconstructed unbiased CV probability distribution, via reweighting of deposited kernels, rather than from the accumulated Gaussian functions used in MetaD.^8^ This approach offers much faster convergence when optimal CVs are employed.^32^ Like other CV-based methods, its efficiency still depends critically on CV quality. To mitigate this limitation, a variant called OPES explore (OPES_E_) was proposed.^33^ Instead of relying on the unbiased probability distribution, OPES_E_ constructs the bias directly from the sampled CV distribution (see Methods), which allows for more dynamic adaptation of the bias and faster exploration of the FEL, albeit at the expense of slow convergence. This makes OPES_E_ particularly attractive for enzymatic systems, where optimal CVs are often difficult, if not unachievable, to define.

Although PathCV and OPES are established methods, their combination in an enzymatic QM/MM context requires nontrivial choices regarding path construction, biasing parameters, trajectory selection, and validation. The present work addresses these issues by providing a workflow that enables efficient exploration of chemically diverse enzymatic reactions while retaining mechanistic interpretability. This approach enables controlled sampling of transitions between metastable states while retaining computational efficiency, allowing multiple reactive events to be captured on QM/MM-accessible time scales. We applied the protocol on three mechanistic distinct enzymes: (i) the polysaccharide lyase *Ps*Alg7A (PL7A),^34,35^ which carries out a β-elimination on mannuronic alginate blocks; (ii) human O-GlcNAcase (OGA),^36,37^ which performs glycosidic bond hydrolysis with retention of configuration; and (iii) the SARS-CoV-2 main protease (MPro),^38,39^ which follows a cysteine protease mechanism. For PL7A, the full single-step reaction was modeled, whereas for OGA and MPro, we focused on their respective rate-limiting steps (Figure 1). We further examine the impact of key OPES_E_ parameters, and compare FELs reconstructed via either direct bias-based estimation or reweighting. Importantly, we introduce a block-selection criterion that provides a robust stopping rule for time-dependent enhanced sampling simulations. Overall, this work delivers a practical protocol for using PathCV-guided OPES_E_ in enzymatic systems, offering straightforward methodological guidance and reliable convergence criteria for QM/MM with enhanced sampling.

**Figure 1:**
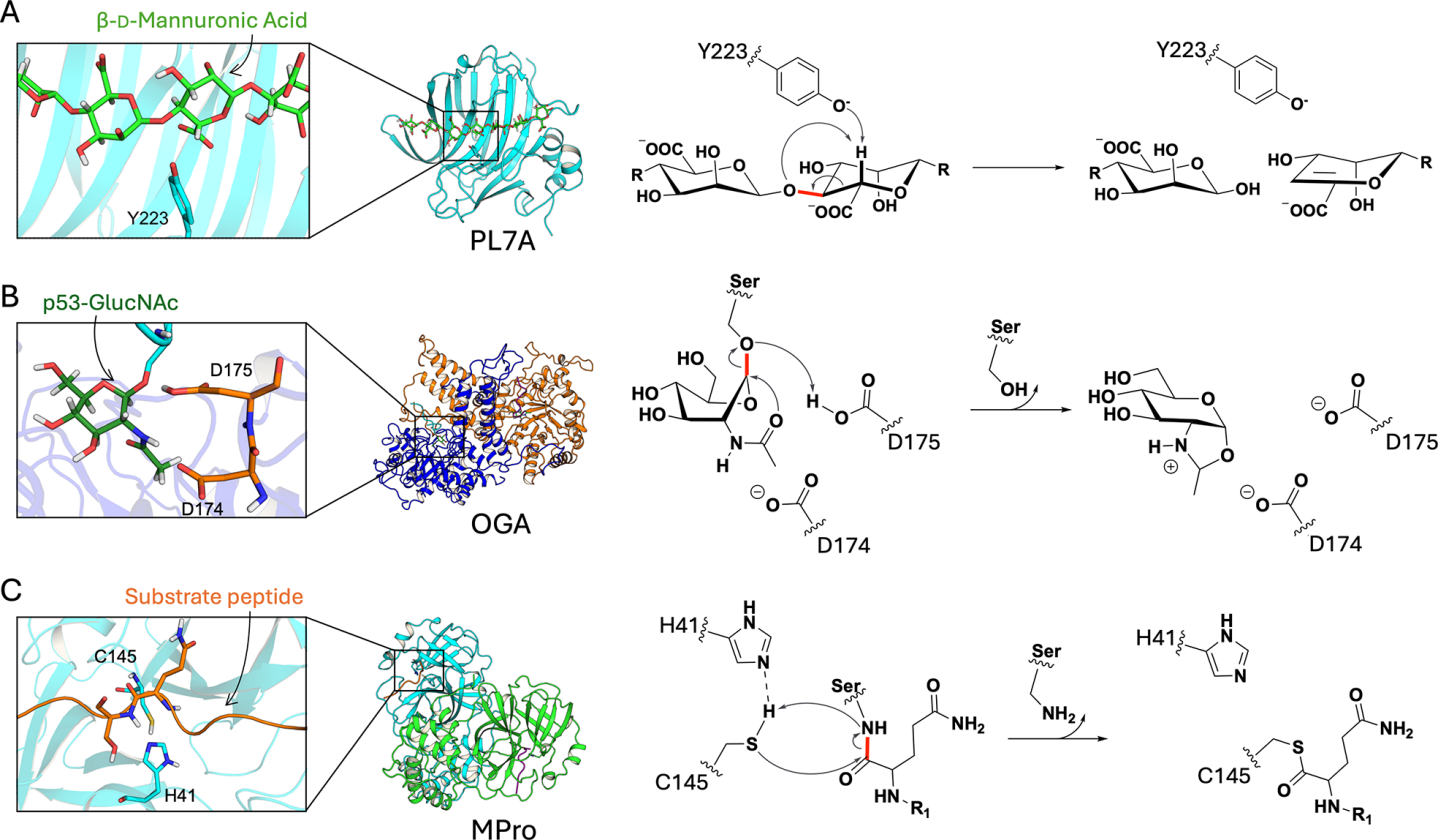
Enzymatic systems investigated in this study. Left panels: cartoon representations of (A) polysaccharide lyase 7A from *Paradendryphiela salina* (PL7A) in complex with the Poly-Mannuronic Acid substrate (PDB ID: 7NCZ); (B) human O-GlcNAcase (OGA) in complex with the glycopeptide p53 (PDB ID: 5UN8); and (C) SARS-CoV-2 main protease (MPro) in complex with an N-terminal autoprocessing substrate (PDB ID: 7MGS), with an active-site close-up shown for each case. Right panels: 2D reaction schemes for each enzyme, with key catalytic distances—corresponding to bonds being broken—highlighted in red.

## 2 Results and Discussion

### 2.1 Construction and Validation of the PathCV

To guide enhanced sampling along meaningful reaction pathways, we first constructed a PathCV for each enzymatic system using a single reactive trajectory connecting the reactant and product basins (Figure 2A and Section 4.2). Unlike the original adaptive formulation of Díaz-Leines and Ensing,^29^ where the path is refined on-the-fly during biased simulations, here the PathCV is generated based on prior simulation data, using a trajectory containing at least one reactive event (i.e., a transition between the two states). The resulting PathCV is then kept fixed and employed in subsequent OPES_E_ runs.

**Figure 2:**
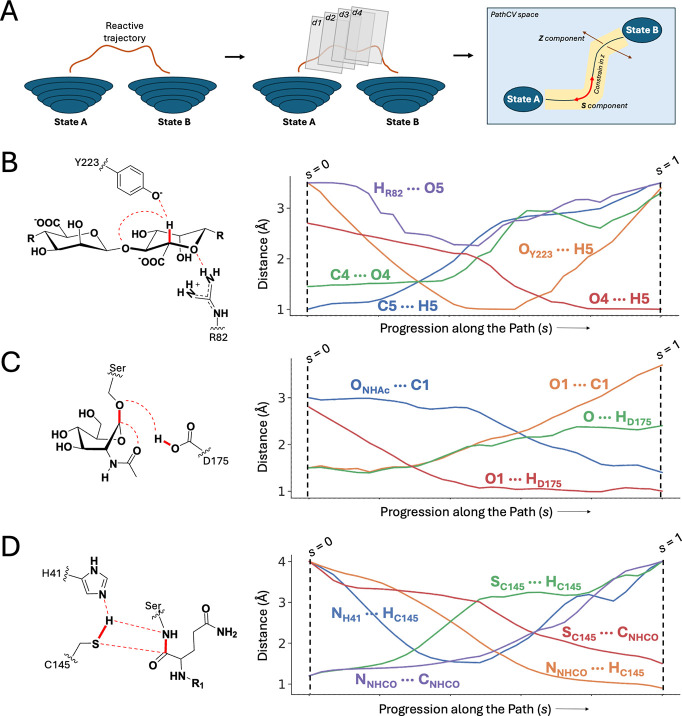
Construction of PathCV from Reactive Trajectories and Descriptor Selection. (A) Schematic of the procedure used to construct a PathCV from preliminary reactive trajectories. The catalytic distances involved in defining the PathCV are shown for (B) PL7A, (C) OGA, and (D) MPro. On the right, each line represents the evolution of a selected distance, with labels color-coded for clarity. On the left, 2D schematic representations of the systems highlight the distances used in PathCV construction. Distances highlighted in red are those chosen to define the PathCV.

The prior reactive trajectories are not required to provide converged free energy profiles or optimal reaction coordinates. Their sole purpose is to capture the qualitative structural rearrangements associated with the chemical step under investigation. This strategy is particularly advantageous compared to the standard implementation in the context of QM/MM simulations, where repeated on-the-fly refinements are computationally prohibitive, and reactive events may be too infrequent to ensure proper path convergence. It should be noted that a similar strategy, employing a fixed initial path for PathCV within the OPES framework, was recently reported by Tiwari and Karmakar (ref 31) to investigate reactions on metal nanoclusters. While our approach shares the philosophy of avoiding computationally expensive on-the-fly path optimization, the present protocol is specifically tailored for enzymatic systems through integration of the OPES_E_ variant and provides a practical approach for data postprocessing.

The selection of descriptors used to construct the PathCV is a critical step in our protocol. Although the optimal choice remains system-dependent and may require adaptation to the specific enzymatic system, a practical approach is to use descriptors associated with bonds that are expected to be be formed and broken during the enzymatic reaction, *i.e.*, using chemically intuitive interatomic distances (Figure 2B–D). The constructed paths show progression of the component *s* along the reactive trajectories. In addition, unbiased simulations of the reactant and product basins showed well-separated and statistically stable distributions along the *s* component (Table S1), confirming that the computed PathCV not only captures the progression of the descriptors along the reaction but also discriminates well between the different metastable states. Together, these tests demonstrate that PathCVs constructed from minimal transition information provide a chemically meaningful and numerically stable reaction-path representation suitable for biasing enhanced sampling at the QM/MM level.

### 2.2 Performance of PathCV-OPES_E_ in Driving Transitions

Having established and validated PathCVs for each system, the performance of OPES_E_ in enhancing exploration between metastable states (reactant and products) was evaluated. In all three enzymes, QM/MM simulations biased along the PathCV using OPES_E_ rapidly generated multiple transitions between the basins within 100 ps of simulation time (Figures 3A–C). PL7A and MPro each exhibited three recrossings, while OGA showed five. A recrossing occurs when the system leaves one metastable state, reaches the other basin, and then returns to the initial state, i.e., crossing the TS twice in the process. Notably, the first transition occurred within approximately 10 ps in all cases, indicating efficient acceleration of barrier crossing.

**Figure 3:**
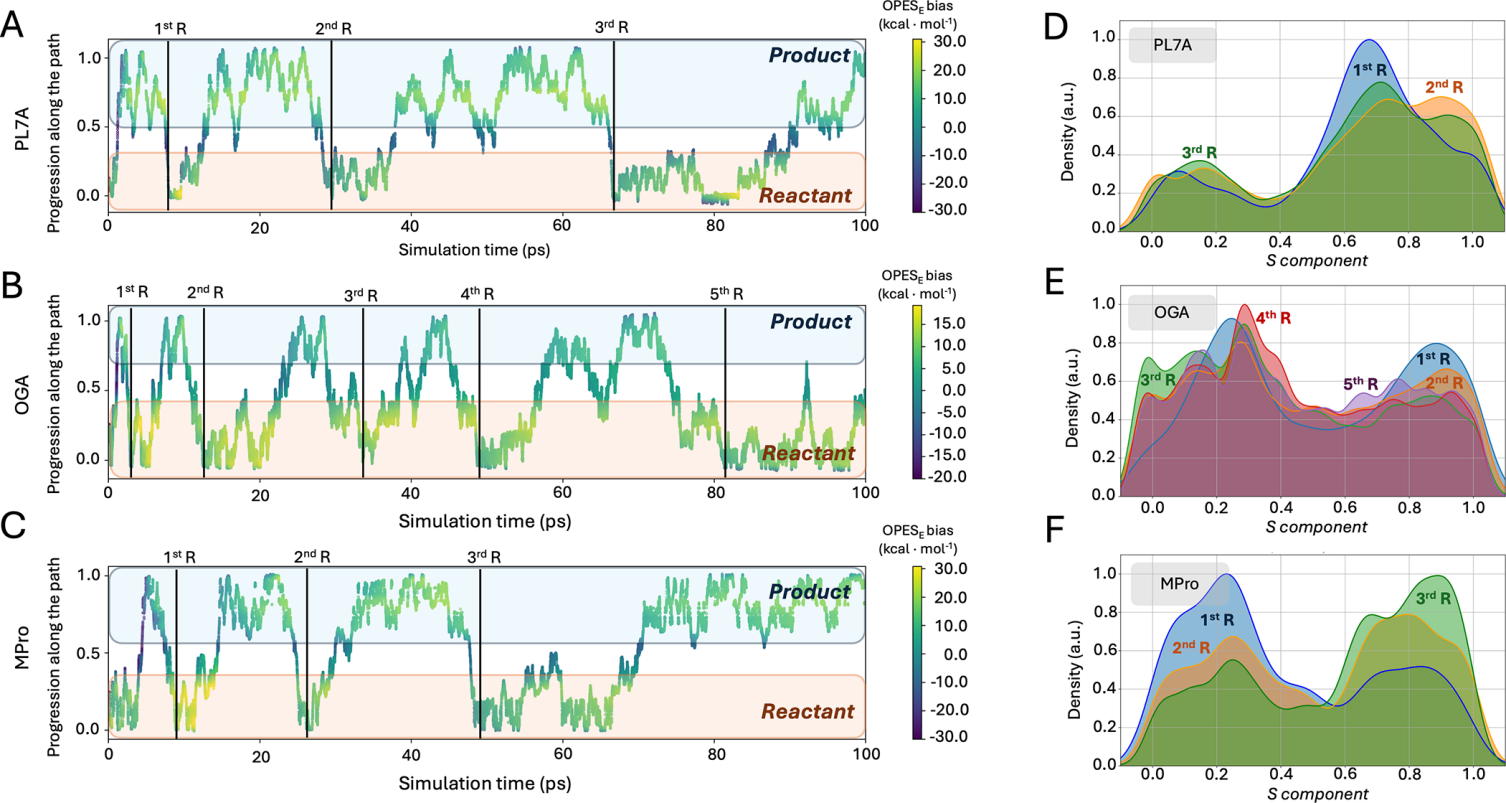
Evolution of the progression along the PathCV in QM/MM OPES_E_ simulations of the enzymatic systems studied. Time evolution of the PathCV *s* component for (A) PL7A, (B) OGA, and (C) MPro. In each graph, shaded regions indicate the reactant and product basins, and black vertical lines mark recrossing events (two transitions) across the TS region. R indicates recrossing over the TS. The simulated time shown is 100 ps. Panels (D)–(F) show probability distributions along the s component of the PathCV sampled during the 100 ps biased simulations for each enzyme. Blue, orange, green, red, and purple curves correspond to probability distributions computed after one, two, three, four, and five recrossing events, respectively.

The above behavior reflects the adaptive bias construction employed by OPES_E_. At early stages of the simulation, broad kernels are deposited along *s*, rapidly filling free energy minima and generating large bias gradients that facilitate escape from metastable basins. As the simulation proceeds and sampling increases, the kernel width decreases, leading to a more refined bias potential and stabilized exploration of the FEL near the TS region (Figure 3D–F).

The trajectories generated during the PathCV–OPES_E_ simulations were further analyzed to assess whether the initial PathCV definition could be improved. To this end, the evolution of the descriptors was averaged along *s* and used to construct a new refined, data-driven PathCV (Figures S1–S4). Analysis of the new and the original PathCV resulted in an almost one-to-one correspondence with the original coordinate (RMSE ≈ 0.02–0.03), along with nearly identical time evolution profiles (Figure S1). In addition, the mean of the individual descriptors at each *s* value closely follows the original input path (Figures S2–S4). These results indicate that the original PathCV already provides a reliable description of the reaction coordinate, making further on-the-fly or iterative optimization unnecessary.

To compare the efficiency of OPES_E_ with other CV-based strategies, additional QM/MM simulations were performed using OPES, MetaD, and WT-MetaD, all employing the same PathCVs (Figures S5–S7). For a fixed simulation length of 100 ps, OPES_E_ consistently produced the highest number of reactive transitions, typically exceeding those observed with the other methods by more than a factor of 2 (Figure 4A). In contrast, MetaD, WT-MetaD, and OPES often generated few or no transitions within the same time window and exhibited substantial variability in the timing of the first reactive event (Figure 2B).

**Figure 4:**
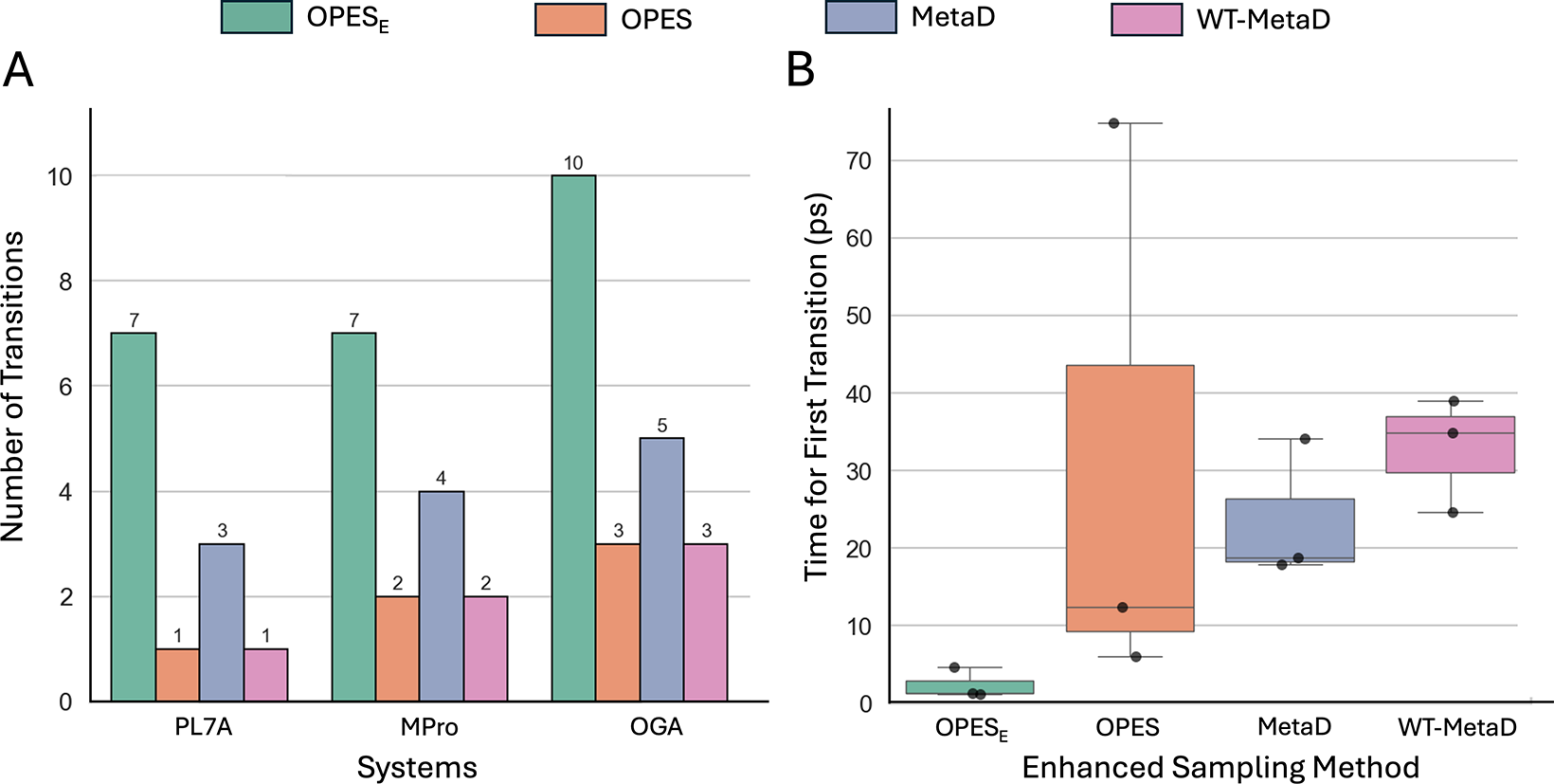
Comparison of CV-based enhanced sampling techniques using the path-based CV to drive transitions between metastable states. (A) Number of transitions observed during QM/MM simulations with the indicated methods. A transition is counted when the system moves from one metastable state to another. (B) Box plots showing the time (in picoseconds) required for the first transition event with each method. Color coding represents the different strategies: green for OPES_E_, orange for OPES, blue for MetaD, and pink for WT-MetaD. Points indicate the measured times for each system.

Together, these results demonstrate that PathCV-guided OPES_E_ provides a robust and efficient strategy for promoting barrier crossings in QM/MM simulations. Its ability to rapidly generate multiple transitions within short simulation time is particularly advantageous for enzymatic systems, where computational cost severely limits accessible time scales. Moreover, the consistent behavior observed across three mechanistically distinct enzymes highlights the utility of OPES_E_ not only as an enhanced sampling method but also as a practical diagnostic tool for assessing the suitability of chosen CVs.

### 2.3 Optimization of OPES_E_ Bias Construction Parameters

Following the validation of OPES_E_ performance in driving reactive transitions, the influence of key bias-construction parameters on sampling efficiency was systematically examined. In the standard OPES_E_ implementation, the probability distribution is built using Gaussian kernels whose width σ is updated dynamically. Two user-defined parameters govern this process: the kernel deposition frequency (PACE) and the adaptive sigma (AS) stride, which controls how often σ is updated (AS = 10 × PACE is the default setup).

To evaluate the impact of these parameters, QM/MM simulations were performed using PACE values of 100, 200, and 500 steps, each combined with AS values of 2×, 5×, and 10× PACE (Table S2), respectively. For each enzymatic system, more than 900 ps of sampling were accumulated. Sampling efficiency was quantified by the number of transitions between reactant and product basins observed within the first 100 ps, providing a consistent metric for comparing parameter sets.

Across all three enzymes, small PACE-AS combinations systematically enhanced sampling efficiency (Figure 5). Specifically, frequent kernel deposition and fast σ updates led to a reasonable number of transitions and accelerated escape from metastable basins. Analysis of the kernel-width evolution confirmed a more rapid decay of σ under these conditions (Figure S8), resulting in a localized and responsive bias. For short QM/MM simulations, this rapid bias adaptation is particularly beneficial, as it facilitates barrier crossing while maintaining directed progression along the reaction coordinate.

**Figure 5:**
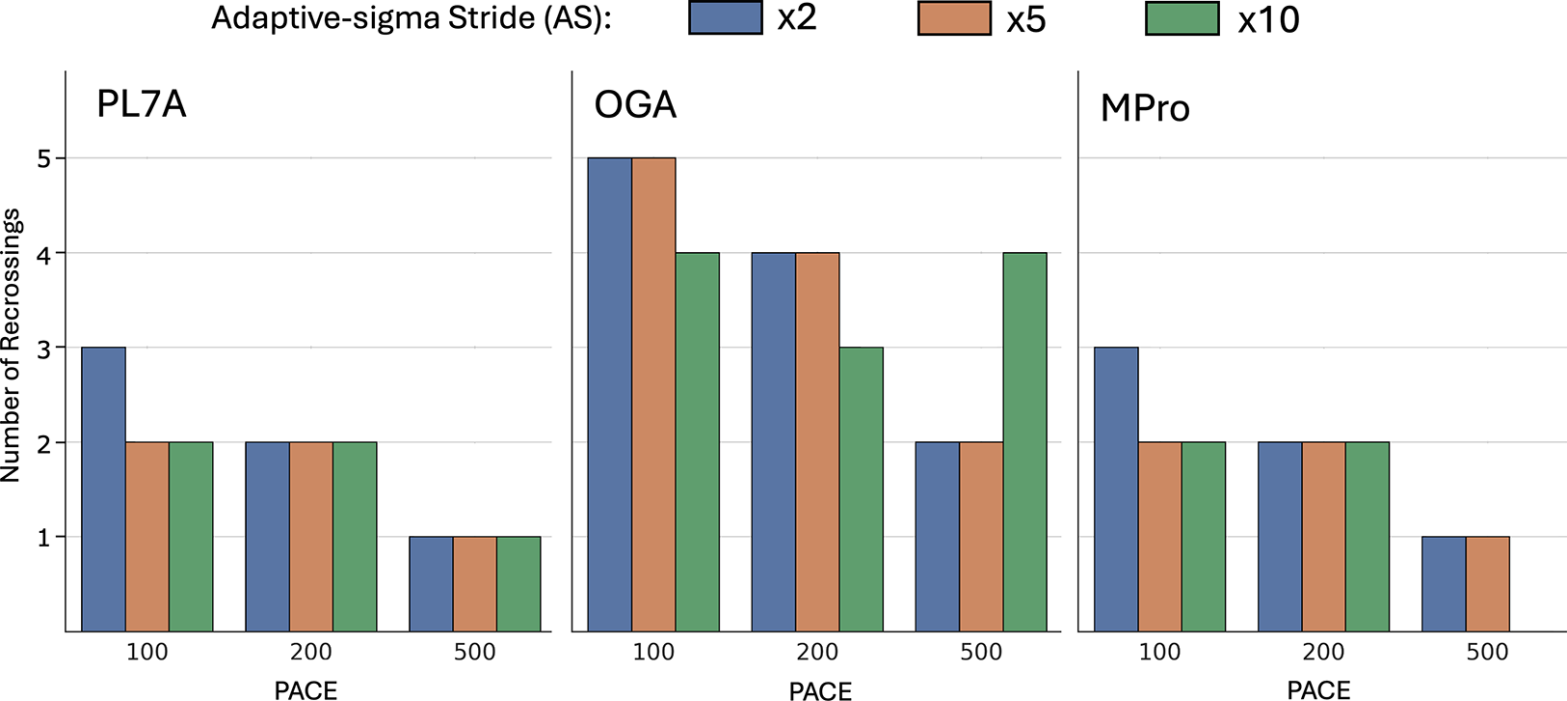
Number of recrossing in the tested systems according to different combinations of OPES_E_ parameters. The *y*-axis shows the number of counted recrossing, while the *x*-axis represents the PACE values used in the simulations. For each PACE value, data is grouped by the adaptive sigma (AS) settings, with AS values corresponding to twice, three times, and five times the PACE represented by blue, orange, and green bars, respectively. The analysis was conducted using 100 ps-long simulations for each combination of PACE and AS.

On the basis of these results, two parameter combinations emerged as optimal compromises between sampling efficiency and computational cost: PACE = 100 with AS = 200 and PACE = 200 with AS = 400. Both settings promote fast yet stable refinement of the bias potential and therefore, can be adopted for FEL reconstruction. This analysis provides practical guidelines for selecting the OPES_E_ parameters in QM/MM studies of enzymatic reactions.

### 2.4 Free Energy Reconstruction

#### 2.4.1 Limitations of Direct FEL Reconstruction

While the generation of multiple reactive transitions is essential for exploring the FELs associated with enzymatic processes, reliable reconstruction of these FELs at the QM/MM level remains challenging. Two reconstruction approaches were evaluated: direct estimation from the accumulated OPES_E_ bias and reweighting-based reconstruction via importance sampling. In principle, both methods should converge to the same FEL given infinitely long sampling. In practice, however, the short time scales accessible to QM/MM simulations and the intrinsically time-dependent nature of OPES_E_ biasing introduce significant discrepancies (Figures S9–S11 and Tables S3–S5).

In particular, early stages of OPES_E_ simulations are dominated by the deposition of broad Gaussian kernels, which can distort the estimated probability distribution and lead to an exaggerated barrier height. This effect is especially sensitive to enzymatic systems, where the FELs are often asymmetric, with one basin significantly more stable than the other. In such cases, the bias must be constructed to overcome the highest barrier along the reaction pathway, which can result in rapid bias accumulation if the system initially samples or becomes transiently trapped in less stable regions.

Directly estimated FELs were especially sensitive to these early fluctuations, while reweighted FELs were still affected by poorly sampled regions, even though smoother estimations were obtained. This behavior is reflected in the Δ*F* convergence analysis (Figures S12–S14). For OGA, where the reactant basin is more stable than the products basin, Δ*F* profiles obtained from the full trajectory and from the postequilibration region are essentially identical, indicating a minor impact of early bias construction. In contrast, for the PL7A and MPro systems, noticeable differences arise, reflecting the stronger influence of early kernel deposition. Across all systems, terminating simulations immediately after a single transition or recrossing resulted in FELs that are inconsistent with experimental observations, highlighting the need for a strategy to identify statistically reliable trajectory segments for free energy reconstruction.

#### 2.4.2 Block Selection Strategy for Robust FEL Estimation

To address the limitations described above, a block selection procedure was implemented to isolate well-equilibrated portions of the trajectory suitable for FEL reconstruction. The approach is inspired by earlier reweighting-based strategies^40^ but is adapted to the dynamic biasing scheme of OPES_E_. Valid trajectory blocks were required to (i) begin in the reactant basin prior to a transition, (ii) contain at least one complete recrossing between reactant and product states, (iii) terminate once both the PathCV component *s* and the bias potential had returned to values comparable to those at the block start, and (iv) occur only after the OPES_E_ reweighting factor, *c*(*t*), had stabilized. The overall workflow, from reaction exploration to block extraction and FEL computation, is summarized schematically in Figure 6A, while the block selection logic is illustrated in Figure 6B.

**Figure 6:**
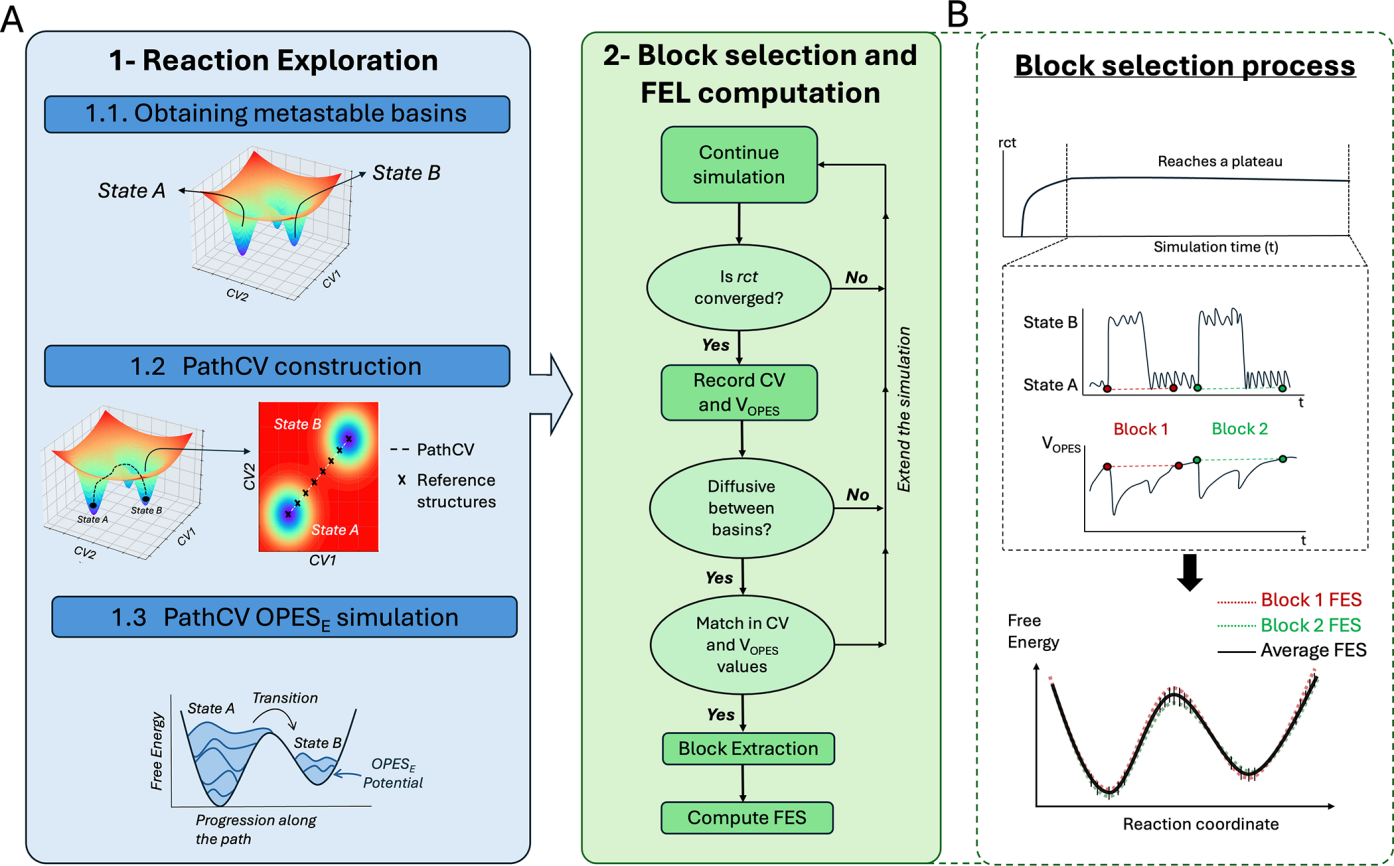
Workflow developed for the analysis of enzymatic reactions. (A) The overall protocol is divided into two main stages: reaction exploration (1) and free energy reconstruction (2). In the first stage (1), an initial hypothesis of the catalytic process is formulated using standard CVs (distances, angles, dihedrals, etc.). An OPES_E_ simulation is performed using geometric descriptors (1.1), and a PathCV is constructed from that preliminary reactive trajectory (1.2). This PathCV is used in subsequent OPES_E_ simulations (1.3). In the second stage (2), the FEL of the process is obtained from the PathCV–OPES_E_ runs, extracting segments or ‘blocks’ from the simulated data according to the procedure illustrated in the scheme. (B) Procedure for block selection, showing the monitoring of key parameters used to define segmentation of the simulated trajectory.

Applying our block-selection criteria, four blocks were identified for OGA and two blocks each for PL7A and MPro in simulations performed with PACE = 100 steps and AS = 200 steps (Figures S12–S14). FELs reconstructed from the selected blocks were highly consistent across independent blocks, exhibiting reduced variability and improved agreement compared with direct and reweighted estimates (Figures S15–S18). Our block-selection strategy produced smoother probability density profiles and more reproducible activation barriers compared to conventional recrossing-based stopping criteria, providing a robust and quantitative framework for FEL reconstruction in QM/MM simulations.

#### 2.4.3 Validation of Enzymatic Mechanisms through Block-Selected FELs

The block-selected FELs enabled detailed mechanistic interpretation of the enzymatic reactions studied (Figure 7). In all cases, the reconstructed FELs allow clear identification of reactant, transition state, and product or intermediate regions along the PathCV, which can be directly associated with representative molecular configurations. Consistent with previous computational and experimental observations (Table S6),^35,37,39^ the reactions were found to be exergonic for PL7A and MPro, whereas in OGA, the intermediate state is energetically higher than the reactant basin (by ∼6 kcal·mol^–1^).

**Figure 7:**
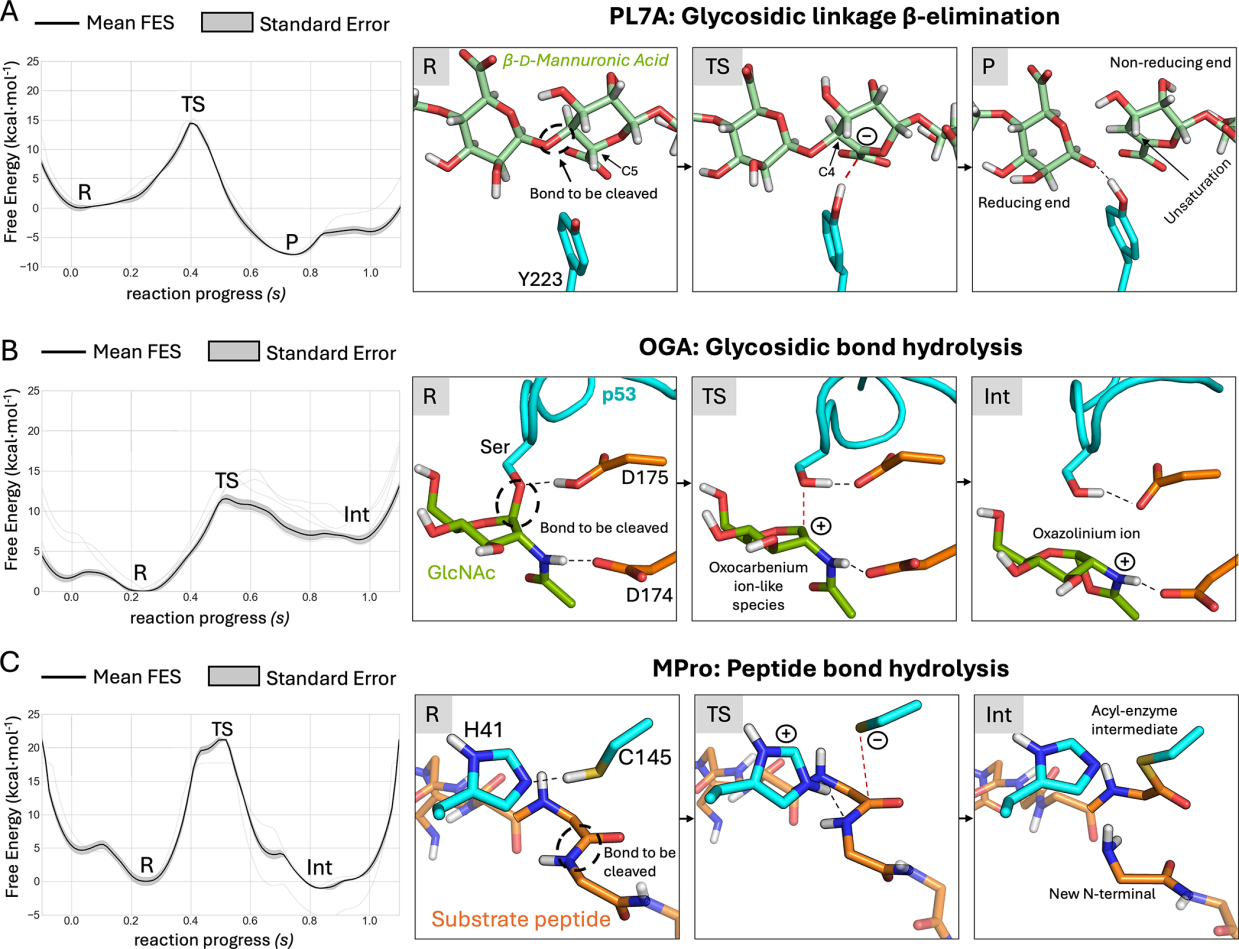
Free energy profiles computed using the block selection criteria. Profiles are shown for (A) PL7A, (B) OGA, and (C) MPro. To the right of each profile, representative molecular configurations of the three main states—reactant, TS, and product/intermediate—along the reaction pathway. Residues are shown as sticks and labeled accordingly. Dashed lines indicate hydrogen bonds (black) and covalent bonds undergoing cleavage (red). The structures correspond to the most representative conformations for each state, as determined by clustering analysis.

To further evaluate the reliability of the reconstructed FELs, we split each valid block into forward and backward reaction segments. Forward segments correspond to trajectories moving from the reactant basin toward products, whereas backward segments describe the reverse passage following the recrossing of the transition region. For each system, chemically relevant catalytic distances were monitored along both directions as a function of PathCV progress coordinate *s*. This analysis was carried out to verify that the reaction mechanism is sampled consistently and to identify possible hysteresis effects arising from the time-dependent bias or from incomplete relaxation of degrees of freedom orthogonal to the PathCV.

##### Alginate Lyase *Ps*Alg7A (PL7A)

For PL7A, forward and backward profiles were examined for the proton abstraction coordinate (C5···H5), the interaction between the catalytic base Y223 and H5 (O_Y223_···H5), and the glycosidic bond cleavage coordinate (O4···C4) (Figure 6A). The C5···H5 and O_Y223_···H5 distances show nearly identical behavior in the two directions, indicating that proton abstraction is reversible and well equilibrated within the sampled blocks. Notably, proton abstraction consistently occurs before cleavage of the glycosidic bond along the PathCV, in line with a concerted but asynchronous β-elimination mechanism.

In contrast, the O4···C4 distance exhibits a small hysteresis in the immediate vicinity of the TS. This deviation remains confined to the transition region and does not extend into the reactant or product basins. Importantly, the relative ordering of the chemical events is unaffected: proton abstraction precedes bond cleavage in both directions (dotted line in Figure 8). This indicates that the mechanistic sequence is robust with respect to the direction of traversal along the reaction coordinate.

**Figure 8:**
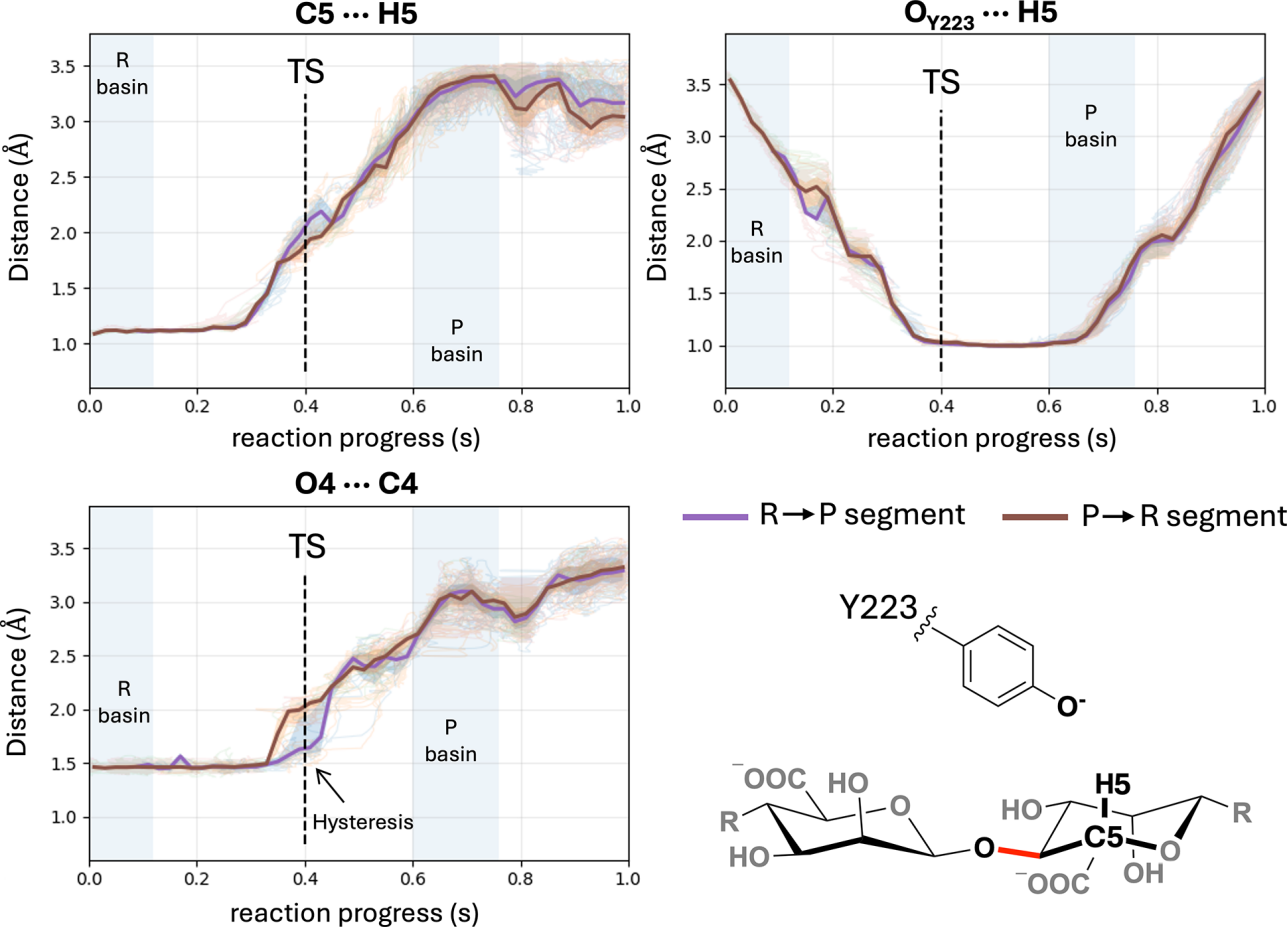
Forward and backward analysis of catalytic distances in PL7A. Forward (purple) and backward (brown) profiles of key catalytic distances plotted as a function of the PathCV component *s* for PL7A, including the proton abstraction coordinates (C5···H5 and O_Y223_···H5) and the glycosidic bond cleavage coordinate (O4···C4). Forward trajectories (purple lines) correspond to reactive passages from reactant to product, while backward trajectories (brown lines) describe the reverse passage following recrossing of the transition region.

##### Human O-GlcNAcase (OGA)

For OGA, the oxazolinium intermediate formation and protonation of the leaving group were analyzed using distance differences as descriptors that capture bond formation and cleavage (Figure 9). Specifically, distances reporting on cyclic oxazolinium formation and proton transfer from D175 to the leaving group were monitored along the forward and backward segments.

**Figure 9:**
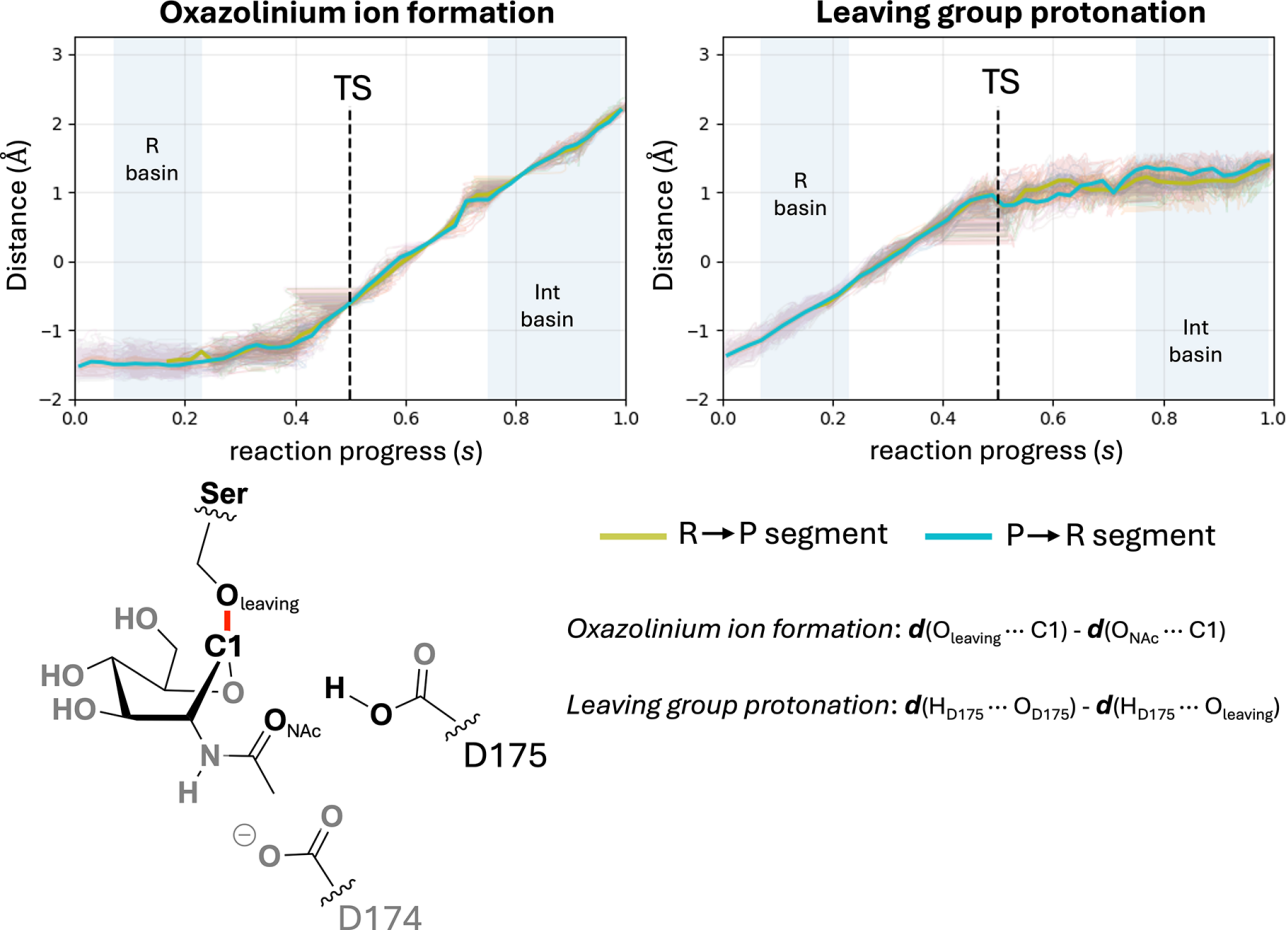
Forward and backward catalytic distance profiles for OGA. Forward (blue) and backward (orange) profiles of reaction coordinates associated with oxazolinium-ion formation and leaving-group protonation in OGA, plotted as a function of the PathCV component *s*. Difference-distance descriptors are used to capture the competition between bond formation and cleavage events.

The resulting distance profiles are nearly superimposable along the entire reaction coordinate including the TS region. No hysteresis is observed, indicating that both proton-transfer and ring-closure steps are sampled consistently within the block-selected trajectories. These results further support a substrate-assisted mechanism involving the formation of a stabilized oxazolinium intermediate, in agreement with the reconstructed FEL and previous mechanistic studies.

##### SARS-CoV-2 Main Protease (MPro)

For MPro, we analyzed the following distances that define the mechanism (Figure 10):

(1) ion-pair formation within the catalytic dyad (proton transfer from C145 to H41; N_H41_···H_C145_).
(2) activation of the nucleophilic cysteine (S_C145_···H_C145_).
(3, 4) polarization (N_peptide_···H_C145_) and cleavage (N_peptide_···C_peptide_) of the scissile peptide bond.
(5) nucleophilic attack by C145 on the carbonyl carbon of the peptide bond (S_C145_···C_peptide_).

**Figure 10:**
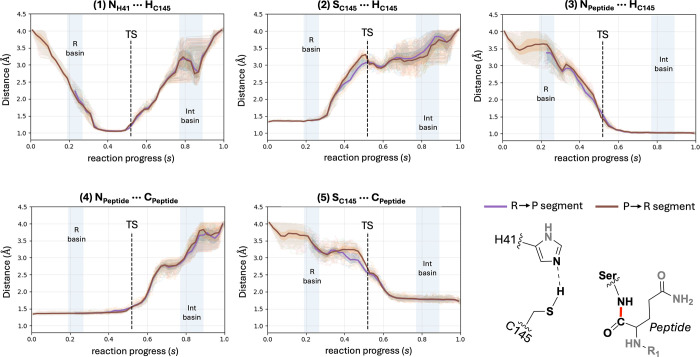
Forward and backward catalytic distance profiles for the SARS-CoV-2 main protease (MPro). Forward (purple) and backward (brown) profiles of distances describing the acylation step in MPro, shown as a function of the PathCV component *s*. The monitored distances report on catalytic dyad ion-pair formation, nucleophilic activation of C145, polarization and cleavage of the scissile peptide bond, and nucleophilic attack.

Both forward and backward profiles overlap closely along *s*, including the TS region associated with ion pair formation. The absence of any hysteresis indicates that the acylation step is sampled consistently in the forward and backward segments of the block selected trajectories. These observations support a mechanism involving transient dyad ion pair formation, followed by nucleophilic attack by Cys145 on the carbonyl carbon atom of the scissile peptide bond, together with proton transfer to the leaving-group amine. These features are consistent with those observed in the reconstructed FEL and previously reported in the literature.^39^

Across all three systems, comparison of forward and backward segments does not reveal any large-scale or systematic hysteresis that would undermine the mechanistic interpretation of the FELs. Particular attention was given to the TS region, which is expected to be the most sensitive part of the reaction coordinate and the most susceptible to hysteresis or other effects. Near the TS, small differences between forward and backward profiles are observed for specific coordinates, most clearly for the glycosidic bond cleavage distance in PL7A (Figure 8). These differences are confined to a narrow range and reflect the intrinsic sensitivity of projecting a high-dimensional system onto a reduced-CV description. Importantly, they do not affect the identification of metastable states or the relative ordering of the underlying chemical events, serving as a practical tool that balances accuracy and computational cost.

Beyond validating the reconstructed FELs, this analysis provides a practical means to assess whether the orthogonal degrees of freedom are sufficiently relaxed along the PathCV and to verify that the reaction pathway is sampled consistently in both directions. It can also help identify undersampled regions or reveal potential alternative reaction pathways.

In case such localized differences are observed, committor analysis provides a natural and rigorous way to characterize the TS ensemble. This approach is commonly applied after sampling the FEL to further characterize TS configurations^35,41^ and could serve as an additional strategy to rule out artifacts from the simulation or the sampling of alternative pathways (including hysteresis). In this context, the forward/backward analysis performed here serves as an efficient diagnostic tool, highlighting regions where more detailed analysis may be needed.

Overall, these results indicate that the PathCV–OPES_E_ protocol, when combined with block-based free energy reconstruction, yields reaction pathways that are sufficiently well-defined for mechanistic interpretation while also highlighting regions where limited sampling may affect the accuracy of the free energy estimates. The ability to identify and rationalize such limitations, and to complement the analysis with committor-based approaches when needed, is convenient for a precise QM/MM analysis of complex enzymatic reactions.

## 3 Conclusions

This work establishes a practical and general protocol for investigating enzymatic reaction mechanisms at the QM/MM level through the combined use of path-based CVs and the OPES_E_-enhanced sampling scheme. By coupling chemically interpretable PathCVs with an exploratory biasing strategy, the approach efficiently promotes reactive transitions between metastable states while remaining compatible with the short time scales typically accessible to QM/MM simulations.

Across three mechanistically distinct enzymatic systems, the PathCV–OPES_E_ protocol accelerated barrier-crossing events and enabled the reconstruction of consistent FELs within limited computational time. Systematic benchmarking against alternative enhanced sampling strategies demonstrated the efficiency and robustness of OPES_E_ when guided by PathCVs in computationally demanding QM/MM settings. Furthermore, analysis of bias construction parameters provided practical guidelines for kernel deposition frequency and adaptive stride, offering insightful recommendations for enzymatic simulations.

We also addressed the challenges of reconstructing free energy profiles from simulations with time-dependent bias potentials. For this purpose, we introduced a block-selection strategy to identify statistically reliable parts of the trajectory for the free energy estimation. This improved the consistency and reproducibility of the resulting profiles, improving mechanistic interpretation and helping to identify potential issues, such as hysteresis, that may require further work.

To further validate the reconstructed FELs, we employed a forward–backward segment analysis. This approach evaluates whether the reaction pathway is sampled across transition events and assesses the adequate sampling of degrees of freedom orthogonal to the PathCV. Close agreement between forward and backward profiles confirms the robustness of the proposed mechanisms, whereas hysteresis or deviations may indicate insufficient sampling or the presence of alternative pathways. Beyond validation, this analysis serves as a practical diagnostic tool for identifying undersampled regions and potential alternative reaction channels. When a more rigorous characterization of TS configurations is required, it can be complemented by committor analysis.

The proposed PathCV–OPES_E_ protocol can also be extended to explore alternative reaction pathways by defining additional PathCVs. This allows different mechanisms to be compared systematically and enables the generation of data sets that can be used in more advanced analysis approaches or computational pipelines for the discovery of enzymatic mechanisms *in silico*. Overall, this framework combines computational efficiency with methodological transparency and chemical interpretability, offering a robust and accessible strategy for both nonexpert users and experienced practitioners aiming to extend sampling and improve FEL estimation.

## 4 Methods

### 4.1 Enhanced Sampling Techniques Used in This Study

In this study, enhanced sampling techniques were employed to accelerate the transitions between the catalytic states of interest along a predefined path-based CV. All of the methods used shared a common principle: introducing a time-dependent bias potential *V*(*s*,*t*) that discourages revisiting previously sampled regions of the CV space, thereby promoting transitions between metastable states and enhancing configurational exploration. The specific construction of this bias, however, differs among the methods.

In conventional **metadynamics (MetaD)**, originally proposed by Laio and Parrinello in 2002,^8^ the bias potential is built as a sum of Gaussian functions periodically added along the trajectory:

$$V\left(s,t\right)=\underset{t^{\prime }<t}{\sum }w\exp\left[-\frac{{\left(s-s\left(t^{\prime }\right)\right)}^{2}}{2\sigma^{2}}\right]$$ 1

where *w* is the Gaussian height, σ its width, and *s*(*t*′) the CV value at deposition *t*′. Over time, *V*(*s,t*) facilitates escape from sampled minima and is expected to converge to the negative of the underlying free energy surface *F*(*s*), up to a constant. However, in standard MetaD, the bias grows linearly with simulation time, which may lead the system to explore unphysically high-energy regions.

To address this limitation, **well-tempered metadynamics (WT-MetaD)** introduces an adaptive scaling of the Gaussian height as the bias accumulates,^42^ ensuring smoother convergence and controlled sampling:

$$w\left(t\right)=w_{0}\exp\left[-\frac{V\left(s\left(t\right),t\right)}{k_{B}\Delta T}\right]$$ 2

where Δ*T* defines the bias factor γ = (*T* + Δ*T*)/*T*. The bias factor can be tuned based on available kinetic information, allowing the system to sample from an effectively scaled free energy surface *F*(*s*)/γ*.*

In contrast, the **On-the-fly Probability Enhanced Sampling (OPES)** method reformulates the bias construction by targeting a desired sampling probability distribution p*­(*s*).^32^ The bias potential is defined as

$$V\left(s\right)=-\frac{1}{\beta }\ln\frac{p^{*}\left(s\right)}{P\left(s\right)}$$ 3

where *P*(*s*) is the instantaneous estimate of the unbiased CV probability distribution and β = 1/(*k*_*B*_ *T*). In practice, *P*(*s*) is iteratively estimated from the trajectory using Kernel Density Estimation:

$$P_{n}\left(S\right)=\frac{\overset{n}{\underset{k=1}{\sum }}w_{k}G\left(s,s_{k}\right)}{\overset{n}{\underset{k=1}{\sum }}w_{k}}$$ 4

where *G*(*s*,*s*_*k*_) represents the kernel function centered at the sampled CV value *s*_*k*_, and each kernel is weighted according to the accumulated bias. This smooth and adaptive update efficiently drives sampling toward p*­(*s*), leading to rapid convergence of the underlying free energy surface.

Finally, the **OPES-explore (OPES**_**E**_ **)** variant shares the same conceptual foundation but modifies the bias update procedure to favor configurational exploration over bias convergence.^33^ Instead of reweighting deposited kernels, the bias is constructed directly from the sampled CV distribution:

$$P_{n}\left(S\right)=\frac{1}{n}\overset{n}{\underset{k=1}{\sum }}G\left(s,s_{k}\right)$$ 5

This formulation enables more dynamic bias adaptation and faster exploration of the CV space, making it particularly suitable for systems in which optimal CVs are difficult to define.

### 4.2 Path Collective Variable (PathCV) Following the Díaz-Leines and Ensing Formulation

We employed the PathCV method developed by Díaz-Leines and Ensing, which constructs a reaction coordinate from a discrete sequence of nodes connecting the reactant and product states in a multidimensional descriptor space. The PathCV returns two variables: (i) a progress component ***s***, which measures position along the path, and (ii) a deviation component ***z***, which measures the perpendicular distance from the path.

#### Representation of Configurations and the Reaction Path

Each molecular configuration is represented as a descriptor vector

$$X=\left(x_{1},x_{2},\cdot \cdot \cdot ,x_{N}\right)$$ 6

and the reaction pathway is approximated by *M* + 1 nodes

$$\left\{s_{0},s_{1},\cdot \cdot \cdot ,s_{M+1}\right\}$$ 7

arranged in order from reactants (*s*_1_) to product (*s*_*M*_). The path is piecewise linear between adjacent nodes.

#### Projection onto the Path: Definition of the Progress Component *s*

For a given configuration *X*, we first identify the closest node *s*_*m*_. The projection of *X* onto the path is then computed using the segment [*s*_*m*_, *s*_*m*+1_]. The fractional coordinate *f* ∈ [0,1] along this segment is obtained analytically from the geometry of the three-node triplet (*s*_*m*–1_, *s*_*m*_, *s*_*m*+1_). In this construction, *f* is the solution of the quadratic expression

$$f=\frac{\sqrt{{\left(v_{1}\cdot v_{3}\right)}^{2}-{∥v_{3}∥}^{2}\left({∥v_{1}∥}^{2}-{∥v_{2}∥}^{2}\right)}-\left(v_{1}\cdot v_{3}\right)}{{∥v_{3}∥}^{2}}$$ 8

with

$$\begin{array}{ccc} v_{1}=s_{m}-X, & v_{2}=X-s_{m-1}, & v_{3}=s_{m+1}-s_{m} \end{array}$$ 9

Once *f* is known, the projected point is

$$Y=s_{m}+f\left(s_{m+1}-s_{m}\right)$$ 10

The unscaled progress coordinate is then

σ=m+f 11

And the final reduced coordinate returned by the PathCV is

$$s=\frac{\sigma }{M}$$ 12

Ensuring that *s* = 0 corresponds to reactant and *s* = 1 to products.

#### Deviation from the Path: Definition of the Distance Component *z*

The second PathCV component is the orthogonal distance between the configuration and its projection:

$$z=∥X-Y∥=∥X-\left[s_{m}+f\left(s_{m+1}-s_{m}\right)\right]∥$$ 13

A small value of *z* indicates that the configuration lies close to the reactive pathway, while larger values reflect excursions into the orthogonal degrees of freedom.

#### Path Generation Using a Reactive Trajectory (GENPATH)

To construct the reference pathway, we used the GENPATH option implemented in PLUMED’s PathCV module. This procedure requires the reactant and product node positions and distributes a user-defined number of intermediate noes between them. In our setup:

- The reactive trajectories connecting reactants to products were used as input,
- The number of middle nodes was set equal to 30 to ensure enough point distribution along the reaction coordinate.
- No additional ‘head’ and ‘tail’ nodes were added beyond the two end points.

The initial guess of the transition path, i.e., linear interpolation between initial and final nodes, was dynamically optimized using the following cycle:

1. For every configuration sampled, i.e., all the MD steps, its displacement from the path is computed and assigned to the two nearest nodes, with weights based on the fractional coordinate *f*.
2. The accumulated displacements for each node define an average correction
$$\Delta s_{j}=\frac{\sum_{k}w_{jk}\Delta z_{\mathrm{jk}}}{\sum_{k}w_{jk}}$$ 14
  where Δ*z_jk_* is the vector from node *j* to configuration *k*.

Node *j* is moved by this average displacement:
$$s_{j}^{\mathrm{new}}=s_{j}^{\mathrm{old}}+\Delta s_{j}$$ 15

The nodes are then reparameterized to maintain equal arc-length spacing along the path.

For PL7A and OGA, previously obtained QM/MM simulations provided suitable reactive trajectories. In the case of MPro, for which no such trajectory was available within our group, a short exploratory OPES_E_ simulation was carried out using simple, chemically intuitive descriptors (i.e., interatomic distances) to identify the transition region. In our calculations, the reference path was refined using information from a transition extracted from the trajectories mentioned above, and the PACE parameter was set equal to the number of MD steps (i.e., frames) corresponding to that transition. This procedure ensured that the initial GENPATH line evolved into a refined pathway closely matching the dynamic transition observed in the reactive trajectory.

### 4.3 Computational Methods: Classical Equilibration, QM/MM Setup, PathCV Construction, and Enhanced Sampling Benchmarks

All systems were first equilibrated at the classical level using Amber20.^43^ Representative equilibrated structures were then used as starting points for QM/MM simulations carried out with CP2K v9.1 ^44^ (see refs 26, 28 and Supporting Information for additional computational details). Reaction mechanisms for PL7A and the MPro had been previously characterized using OPES_E_, while the mechanism for OGA was explored using metadynamics (MetaD). The reactant and product basins identified in these prior studies served as reference states for constructing the PathCVs employed here.

PathCVs were defined using a set of interatomic distances as descriptors (Figure 2B–D). Five distances were used for PL7A and MPro and four for OGA. For each enzyme, unbiased QM/MM *NVT* simulations (10 ps) were performed starting from both the reactant and the product configurations to ensure local equilibration and to extract statistical descriptors for basin definitions (Table S1). The resulting PathCV consisted of two components: (i) the progress component *s*, used as the biased CV, and (ii) the perpendicular deviation component *z*, which was not biased directly but restrained by an upper wall potential at 0.65 a.u. to prevent the exploration of geometrically irrelevant regions of the CV space. Path construction was performed using GENPATH, refined using the on-the-fly path-update procedure (see Section 4.2), and kept fixed in subsequent simulation.

To assess the ability of these PathCVs to drive reactive transitions, we performed enhanced sampling simulations using metadynamics (MetaD), well-tempered metadynamics (WT-MetaD), OPES, and the OPES_E_ variant. Gaussian hills for MetaD and WT-MetaD were deposited with a width of 0.01 and height of 1.0 kcal·mol^–1^. WT-MetaD simulations used a bias factor of 50 for PL7A and MPro and 34 for OGA. OPES and OPES_E_ simulations were conducted with target free energy barriers of 30 kcal·mol^–1^ for PL7A and MPro and 20 kcal·mol^–1^ for OGA. In all cases, the external bias was applied exclusively to the *s* component of the PathCV, while the *z* component was constrained as described above.

After confirming that the combination of PathCV and OPES_E_ induced transitions between metastable states, we systematically examined how OPES_E_ parameters affect sampling efficiency in PathCV space. Specifically, we explored three PACE values (100, 200, and 500 MD steps, corresponding to 0.05, 0.1, and 0.25 ps) and three adaptive sigma stride values (2×, 5×, and 10× PACE), giving nine parameter combinations per enzyme. For each combination, a QM/MM OPES_E_ simulation of at least 100 ps was performed, yielding a cumulative simulation time exceeding 900 ps per system (Table S2).

### 4.4 FEL Calculation Based on Block Selection and Comparative Analysis

QM/MM simulations are computationally demanding, often limiting the extent of CV-space exploration and, consequently, the accuracy of the FEL estimation. Although OPES_E_ efficiently promotes transitions between metastable states, the resulting FEL can depend on the simulation stopping point since the bias potential may continue to evolve when new regions of CV space are sampled. To improve statistical consistency, we implemented a block selection and analysis protocol (Figure 5) that extracts event-containing trajectory segments in which the bias is locally stabilized and reconstructs and computes FELs from smoothly reconstructed probability distributions.

The block-localized reconstruction used here was inspired by previous reweighting formalisms developed for metadynamics. Bonomi et al. introduced *c*(*t*) as a time-dependent bias offset that relates the biased probability distribution to the unbiased Boltzmann distribution and showed that its time derivative is equal to the negative expectation value of the time derivative of the bias,

$$ċ\left(t\right)=-{\left\langle \mathrm{V̇}\left(s,t\right)\right\rangle }_{P\left(s,t\right)}$$ 16

Thus, when *ċ*(*t*) is small (ideally zero), the expectation value of the time derivative of the bias is also small, indicating that the biased distribution is approximately stationary over the considered interval.^45^ Tiwary and Parrinello later showed that the time-dependent constant associated with metadynamics can be used to construct a time-independent and locally convergent free energy estimator, allowing the quality of convergence to be assessed locally in CV space.^40^ Motivated by these ideas, we used the apparent plateau of the OPES_E_ bias offset *c*(*t*) as an indicator of small bias deviations over time, together with a start/end bias-closure criterion, to identify rare-event blocks for locally quasi-static FEL estimation.

#### Block Selection Procedure

The protocol comprises four steps:

##### 1. Bias-Offset Plateau Identification

***-*** After the initial transitions (Figure 2), the evolution of the OPES_E_ time-dependent bias offset *c*(*t*) was monitored. Early, highly fluctuating regions were discarded. The analysis started only after *c*(t) reached an apparent plateau, indicating that the bias was evolving slowly enough over the selected interval to be treated as locally quasi-static.

##### 2. Block Start Definition

**-** The CV space was divided into reactant and product basins based on unbiased NVT simulations (Table S1). Basin centers were determined by the mean (μ) of the PathCV progress component *s*, with limits set at μ ± 1σ. The first frame in the reactant basin preceding a successful transition event was defined as the block starting point.

##### 3. Block End Definition

**-** Two convergence conditions were required:

(i) the sampled *s* value must span the entire transition region between reactant and product basins, and
(ii) the end point coordinate *s*_*b*_ must return to the starting basin (reactant basin), and the bias-closure condition

$$\left|V\left(s_{b},t_{b}\right)-V\left(s_{a},t_{a}\right)\right|\leq k_{B}T$$ 17

must be satisfied, where (*s*_*a*_, *t*_*a*_) and (*s*_*b*_, *t*_*b*_) are the block start and end points.

In the ideal case, *s*_*b*_ = *s*_*a*_, so the criterion measures whether the local bias at the same CV point remains within a single thermal oscillation over the selected interval. The first frame fulfilling both conditions was used to define the block end point.

##### 4. Block Extraction, Preprocessing, and FEL Reconstruction

- Trajectory segments satisfying the above criteria were extracted using the MLCOLVAR Python library (*load_frame* function).^46^ To ensure uniform sampling along the CV axis, the data were regridded onto a continuous one-dimensional grid. The s-values were interpolated using cubic spline interpolation (*scipy.interpolate.interp1d*)^47^ to produce a uniformly spaced array of *N*_grid_ points (500.000.000 points used in this study). This step guarantees consistent kernel overlap in the subsequent KDE calculation, reducing numerical artifacts arising from uneven time sampling in the biased trajectories.

The FEL is conventionally computed from the importance-sampling reweighting distribution:^33^

$$F\left(s\right)=-\frac{1}{\beta }\log\underset{k}{\sum }e^{\beta V_{k-1}\left(s_{k}\right)}G\left(s,s_{k}\right)$$ 18

where *G*(*s*,*s*_*k*_) is a Gaussian kernel centered at the sampled point *s*_*k*_, and *V*_*k*–1_(*s*_*k*_) denotes the OPES_E_ bias evaluated at *s*_*k*_ using the bias estimate available immediately before sample *k* was collected.

For rare-event analysis, FEL reconstruction was restricted to selected event-localized blocks $B=\left\{1,\cdot \cdot \cdot ,N_{B}\right\}$ . Each block *b* is defined by the Heaviside window

$$W_{b}\left(t_{k}\right)=H\left(t_{k}-t_{b}^{\mathrm{start}}\right)-H\left(t_{k}-t_{b}^{\mathrm{end}}\right)$$ 19

which yields the event-localized estimator

$$F_{b}\left(s\right)=-\frac{1}{\beta }\log\;\underset{k}{\sum }e^{\beta V_{k-1}\left(s_{k}\right)}G\left(s,s_{k}\right)W_{b}\left(t_{k}\right)$$ 20

and the exponential average is reported as

$$\overset{¯}{F}(s)=-k_{B}Tln\left[\frac{1}{N}\underset{b}{\sum }e^{-F_{b}(s)/k_{B}T}\right]$$ 21

Because all blocks originate from the same OPES_E_ trajectory, they are not treated as fully independent simulations, but as approximately independent block estimates after satisfying the local stationarity and closure criteria. Thus, a combined estimator may be given as

$$F_{B}\left(s\right)=-\frac{1}{\beta }\log\;\underset{k}{\sum }e^{\beta V_{k-1}\left(s_{k}\right)}G\left(s,s_{k}\right)W_{B}\left(t_{k}\right)$$ 22

with

$$W_{B}\left(t_{k}\right)=\overset{N_{B}}{\underset{b=1}{\sum }}W_{b}\left(t_{k}\right)$$ 23

The bandwidth parameter *h* for the kernel function was computed using Silverman’s rule of thumb:

$$h={\left(\frac{4\sigma^{5}}{3n}\right)}^{1/5}$$ 24

where σ is the standard deviation of the CV in each block and *n* is the number of effective samples. KDE was implemented via the *compute_fes* routine from MLCOLVAR,^46,48^ using reweighting factors $w_{i}=e^{V_{i}/k_{B}T}$ .

#### Forward and Backward Analysis

To assess the directional consistency of the reconstructed FELs and identify possible hysteresis effects, each extracted block was further decomposed into *forward*- and *backward*-reaction segments. Forward segments correspond to trajectory portions progressing from the reactant basin toward products, while backward segments describe the reverse passage following the recrossing of the transition region.

For each system, chemically relevant descriptors were selected based on the basic reaction mechanism and monitored as a function of the PathCV progress coordinate *s* along both the forward and backward segments. Distance data were projected onto the same interpolated *s*-grid used for FEL reconstruction, allowing direct comparison between directions. Mean profiles and variability were computed independently for forward and backward segments within each block, enabling the identification of local differences, particularly in the vicinity of the TS region.

This forward/backward analysis serves as a diagnostic tool to evaluate the sampling of orthogonal degrees of freedom and to identify regions where projection onto a reduced CV description may introduce discrepancies.

#### Implementation Details

All code for block identification, interpolation, and FEL computation was implemented in Python 3.10, using NumPy,^49^ SciPy,^47^ and MLCOLVAR libraries.^46^

## Supplementary Material



## Data Availability

All the simulation data and the Jupyter-notebook employed in this study can be found at the following zenodo DOI: 10.5281/zenodo.14206227.

## Notes

Methodology description of MPro system simulations; PathCV validation and descriptor evolution along the *s* component during PathCV–OPES_E_ simulations for the PL7A, OGA, and MPro systems, PathCV coupled with enhanced sampling methods evolution across different systems, evolution of sigma decay in different OPES_E_ simulations, free energy profiles computed after recrossing events in the different systems, FEL comparison with- and without discarding initial simulation data, analysis of the probability densities computed after recrossing events and following the block selection approach, QM region with CV definition for the MPro system, preliminary Mpro system OPES_E_ simulation with canonical CV; and tables showing statistic from the system basins, summary of the simulation performed in this study, energetic parameters extracted from the computed FELs, and reference values from prior studies (PDF)
