# Supplementary material for: Efficient QM/MM Modeling of Enzymatic Reactions Combining PathCV with OPES
Source: J Chem Theory Comput. 2026 May 27;22(11):5867–81. doi: 10.1021/acs.jctc.6c00492 (PMC13255176; doi:10.1021/acs.jctc.6c00492)
Supplement: Supplementary file 1 [file ct6c00492_si_001.pdf]

Supporting Information

Efficient QM/MM Modeling of Enzymatic Reactions combining PathCV with OPES

José Pablo Rivas-Fernández[1,*], Martin Calvelo[1], Mert Sagiroglugil[1], Qinghua Liao[1] and Carme Rovira[1,2,*]

1 Departament de Química Inorgànica i Orgànica & IQTCUB, Universitat de Barcelona, Martí i Franquès 1, 08028 Barcelona, Spain
2 Institució Catalana de Recerca i Estudis Avançats (ICREA), Passeig Lluís Companys, 23, 08020 Barcelona, Spain

Co-corresponding author e-mail: jprivas@ub.edu, c.rovira@ub.edu

Contents

SI Methods

Classical molecular dynamics (cMD) simulations of the MPro system.

QM/MM molecular dynamics simulations of the MPro system.

QM/MM OPES-Explore simulations of the MPro system.

Supplementary Figures

Figure S1. PathCV validation.

Figure S2. Descriptor evolution along the s component during the PathCV-OPES$_E$ runs in the PL7A system.

Figure S3. Descriptor evolution along the s component during the PathCV-OPES$_E$ runs in the OGA system.

Figure S4. Descriptor evolution along the s component during the PathCV-OPES$_E$ runs in the MPro system.

Figure S5. Evolution of the bias potential and cumulative number of transitions for each enhanced sampling method in simulations of the PL7A system.

Figure S6. Evolution of the bias potential and cumulative number of transitions for each enhanced sampling method in simulations of the OGA system.

Figure S7. Evolution of the bias potential and cumulative number of transitions for each enhanced sampling method in simulations of the MPro system.

Figure S8. Evolution of sigma decay in the PathCV-$OPES_E$ simulations for different systems.

Figure S9. FELs computed after recrossing events for the PL7A system.

Figure S10. FELs computed after recrossing events for the OGA system.

Figure S11. FELs computed after recrossing events for the MPro system.

Figures S12. Block-selection strategy and free energy convergence analysis for the PL7A system.

Figures S13. Block-selection strategy and free energy convergence analysis for the OGA system.

Figures S14. Block-selection strategy and free energy convergence analysis for the MPro system.

Figure S15. FELs reconstructed from block-selected trajectories with and without discarding initial bias-stabilization frames.

Figure S16. Probability density analysis for the PL7A system using different splitting methods.

Figure S17. Probability density analysis for the OGA system using different splitting methods.

Figure S18. Probability density analysis for the MPro system using different splitting methods.

Figure S19. QM region and collective variable definition used in the QM/MM simulations of the MPro system.

Figure S20. Time evolution of relevant distances during the QM/MM $OPES_E$ simulations.

Supplementary Tables

Table S1. Gaussian distribution parameters used to define MC and P states for block extraction.

Table S2. Summary of all simulations performed in this study and corresponding $OPES_E$ parameters.

Table S3. Energetic parameters extracted from FELs after recrossing events for the PL7A system.

Table S4. Energetic parameters extracted from FELs after recrossing events for the OGA system.

Table S5. Energetic parameters extracted from FELs after recrossing events for the MPro system.

Table S6. Reference activation barriers and free energy differences obtained from previous computational and experimental studies.

References

SI Methods

Classical Molecular Dynamics (cMD) simulations of Sars-CoV-2 main protease.

We used the crystal structure of SARS-CoV2 (PDB ID: 7MGS)[1] as the starting structures for all simulations reported in this work. The mutation of the catalytic residue (C145A) was mutated back with Chimera.[2] The protonation states of His were chosen based on hydrogen bond network manually checked with Chimera. The simulations were performed at pH 7.0, thus all Asp and Glu residues were negatively charged while all Arg and Lys residues were positively charged. The complex system was placed in the center of a cubic box 110×110×110 Å^3 with a distance of at least 10 Å between the surface of the solute and the edge of the box. The box was then solvated with TIP3P water molecules, and counterions were added to neutralize the system and achieve a concentration of NaCl of 0.15 Mol/L. The protein was described using the Amber ff14SB force field.[3] The LEaP module of AMBER 20 was used to generate the topology and coordinate files for the classical MD simulations,[4] which were carried out using the CUDA version of the PMEMD module[5] of the AMBER 20 simulation package. The solvated system was first subjected to 5000 steps steepest descent minimization, followed by 5000 steps conjugate gradient minimization with positional restraints on all heavy atoms of the solute, using a 100 kcal mol^{-1} Å^{-2} harmonic potential. The minimized system was then heated up to 300 K using the Berendsen thermostat, with a time constant of 1 ps for the coupling, and 100 kcal mol^{-1} Å^{-2} positional restraints applied over three 500-ps steps of heating process. The positional restraints were then gradually decreased to 5 kcal mol^{-1} Å^{-2} over five 500-ps steps of NPT equilibration, using the Berendsen thermostat and barostat to keep the system at 300 K and 1 atm. For the production runs, each system was subjected to 500 ns of sampling in an NPT ensemble at constant temperature (300 K) and constant pressure (1 atm), controlled by the Langevin thermostat, with a collision frequency of 2.0 ps^{-1}, and the Monte Carlo barostat with a coupling constant of 1.0 ps. The SHAKE algorithm was applied to constrain all bonds involving hydrogen atoms.[6] A cut-off of 10 Å was applied to all non-bonded interactions, with the long-range electrostatic interactions being treated with the particle mesh Ewald (PME) approach. A time step of 2 fs was used for all the classical simulations, and coordinates were saved from the simulation every 10 ps. Three independent runs were performed.

QM/MM MD simulations

One representative snapshot extracted from the classical MD simulations was used for the subsequent QM/MM MD simulations,[7,8] which combines Born-Oppenheimer MD simulation, based on density functional theory (DFT), with force-field MD methodology. The QM region consists of residues H41 and C145 as well as parts of the substrate peptide, resulting in a total

number of 34 QM atoms (including 6 capping hydrogens), as showed in Figure S19. The dangling bonds between the QM and MM region were capped with hydrogen atoms.[9] The QM region was enclosed in an isolated supercell of size 14.0 × 14.0 × 14.0 $Å^3$. All QM/MM MD simulations were performed using CP2K v9.1[10] interfaced with PLUMED v2.8.[11] The QM region was treated at the DFT (BLYP) level, employing the dual basis set of Gaussian and plane-waves (GPW) formalism, whereas the remaining part of the system was modelled at the classical level using the same parameters as in the classical MD simulations. The Gaussian triple-ζ valence polarized (TZV2P) basis set was used to expand the wave function, while the auxiliary plane-wave basis set with a kinetic energy cut-off of 400 Ry and GTH pseudopotentials was utilized to converge the electron density.[12] Initially, the system was minimized using LBFGS method[13] for 1000 steps, then the minimized system was subject to a 10 ps equilibration without any restraints under NVT ensemble controlled by a velocity rescaling thermostat[14] with a coupling time constant of 10 fs and an integration time step of 0.5 fs.

QM/MM OPES-explore simulation

After QM/MM equilibration, we then performed OPES-explore (on-the-fly probability enhanced sampling, exploration mode, we refer it as $OPES_E$)[15] simulation to explore the enzymatic reaction, so that we can use the obtained trajectory to train a path collective variable (PathCV) for further simulations.[16,17] With $OPES_E$, the biasing potential is built through the sampled CV probabilities, which are represented by Gaussian kernels. As shown in Figure S19, two distance differences were used as the two collective variables (CVs) in the simulation, CV1 is used to describe the proton transfer at the three sites (Nε2 of H41, NH2 of S6', Sγ of C145) while CV2 is accounting for the nucleophilic attack to the carbonyl group (C=O of Q5') by deprotonated $S\gamma^{1-}$ of C145 and peptide bond cleavage. The BARRIER parameter was set to 30.0 kcal/mol, which was used to build the targeted probability distribution of the two CVs. Kernels were added every 100 steps (50 fs) to update the sampled probability of the two CVs, which also means that the biasing potential was updated every 100 steps to push the system to sample unvisited region. Two recrossing over the transition state were observed in the simulation (Figure S20), which is necessary to accumulate enough data to train a reasonable PathCV. from where a free energy landscape was obtained for the catalyzed reaction.

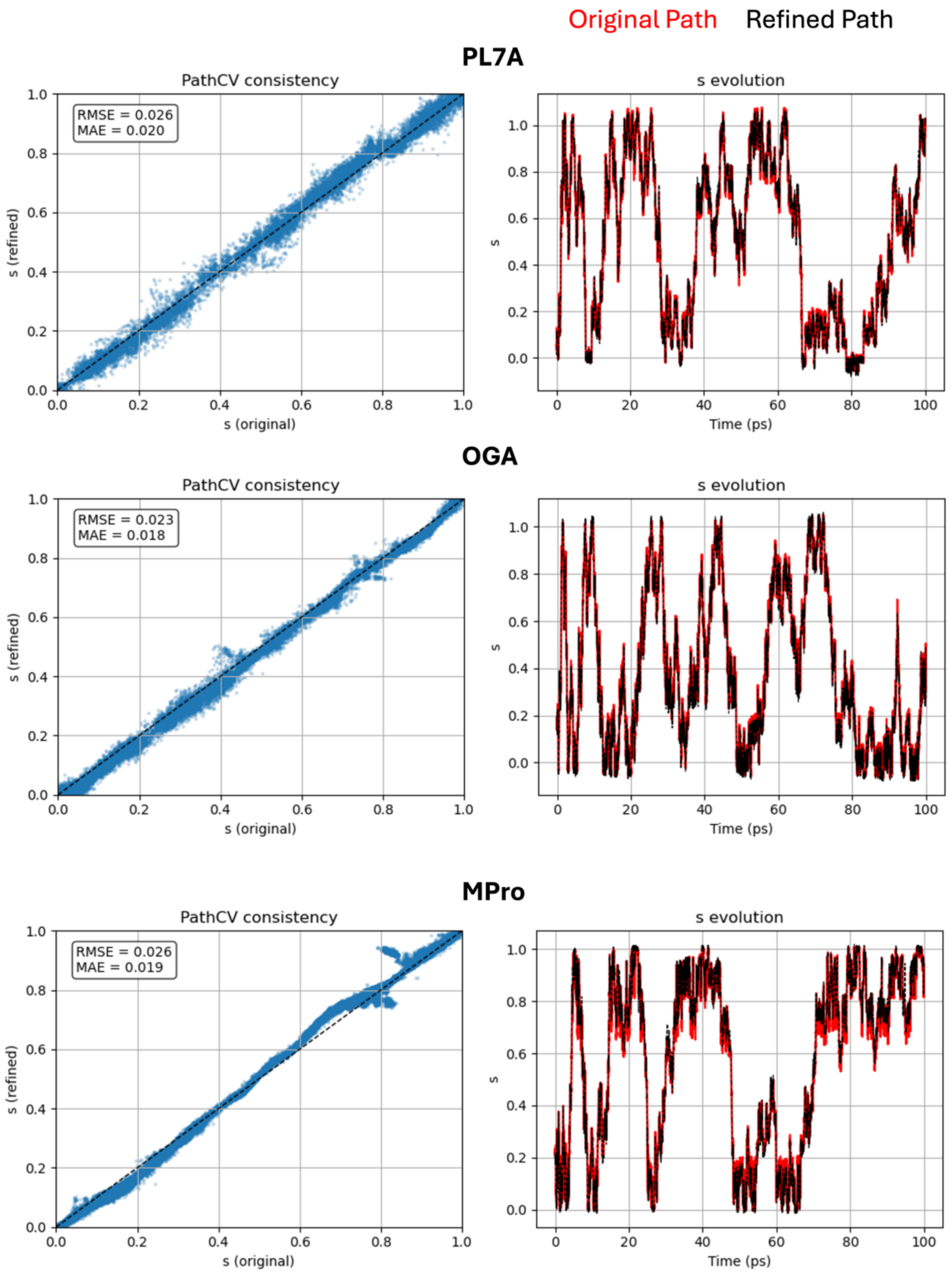

Figure S1. Consistency between original and refined PathCV definitions across the three systems (PL7A, OGA, and MPro). Left panels show the correlation between the progress coordinate *s* computed using the original and refined paths, with the dashed line indicating perfect agreement. The low RMSE and MAE values demonstrate a near one-to-one correspondence between both definitions. Right panels compare the time evolution of *s* along the trajectories, showing overlapping profiles for the original (red) and refined (black) paths.

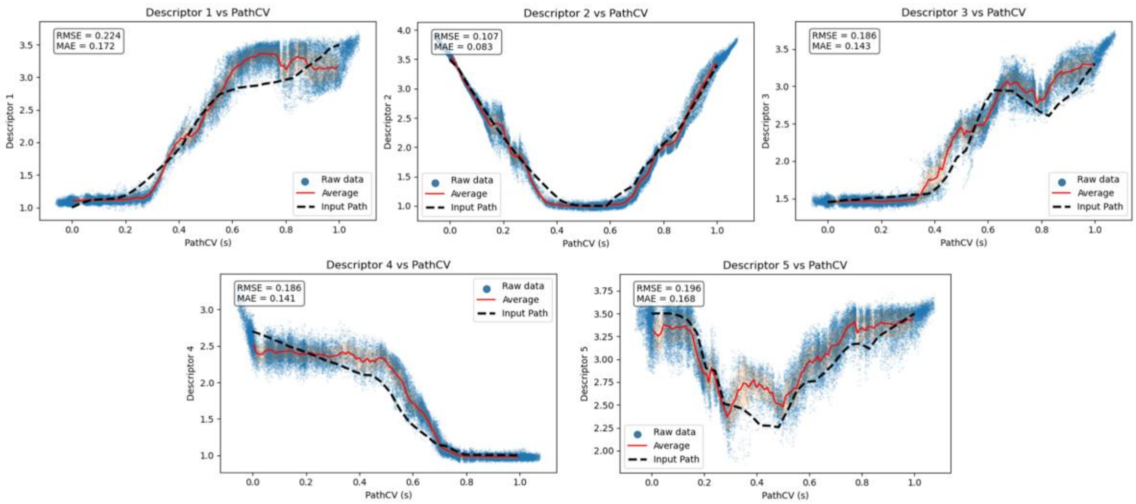

Figure S2. Evolution of descriptors along the PathCV for the PL7A system. Each panel shows the distribution of a given descriptor as a function of the PathCV progress coordinate (*s*). Blue points represent raw simulation data, the red line corresponds to the average profile, and the dashed black line indicates the original input path used to define the PathCV. The close agreement between the averaged profiles and the input path, together with the relatively low RMSE and MAE values, indicates that the initial PathCV definition captures the main catalytic trends. Data were extracted from simulations performed using the exploratory variant of OPES (PACE = 100, AS = 200).

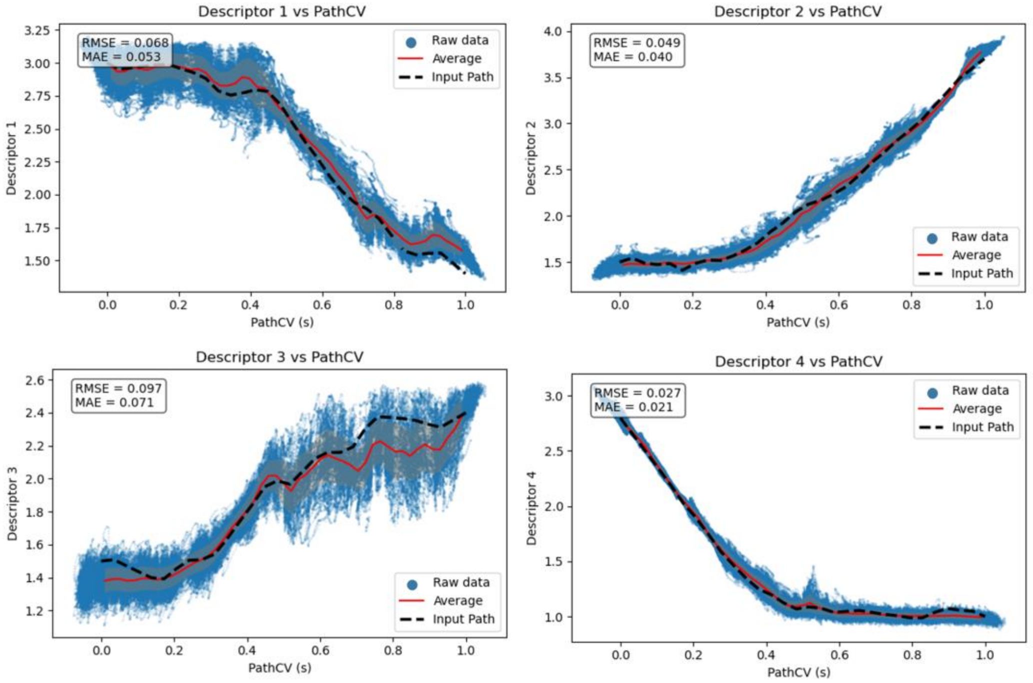

Figure S3. Evolution of descriptors along the PathCV for the OGA system. Each panel shows the distribution of a given descriptor as a function of the PathCV progress coordinate (*s*). Blue points represent raw simulation data, the red line corresponds to the average profile, and the dashed black line indicates the original input path used to define the PathCV. The close agreement between the averaged profiles and the input path, together with the relatively low RMSE and MAE values, indicates that the initial PathCV definition captures the main catalytic trends. Data were extracted from simulations performed using the exploratory variant of OPES (PACE = 100, AS = 200).

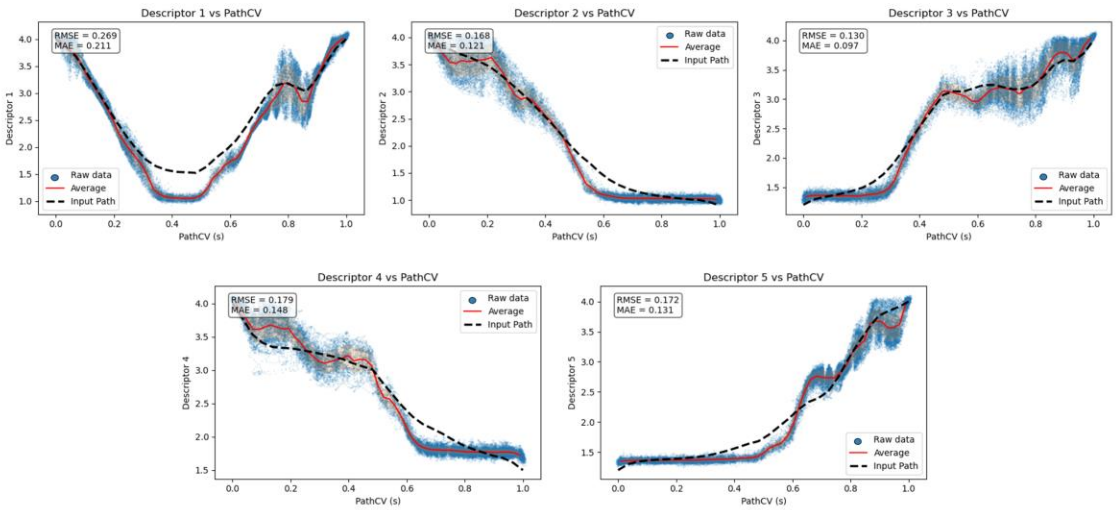

Figure S4. Evolution of descriptors along the PathCV for the MPro system. Each panel shows the distribution of a given descriptor as a function of the PathCV progress coordinate (s). Blue points represent raw simulation data, the red line corresponds to the average profile, and the dashed black line indicates the original input path used to define the PathCV. The close agreement between the averaged profiles and the input path, together with the relatively low RMSE and MAE values, indicates that the initial PathCV definition captures the main catalytic trends. Data were extracted from simulations performed using the exploratory variant of OPES (PACE = 100, AS = 200).

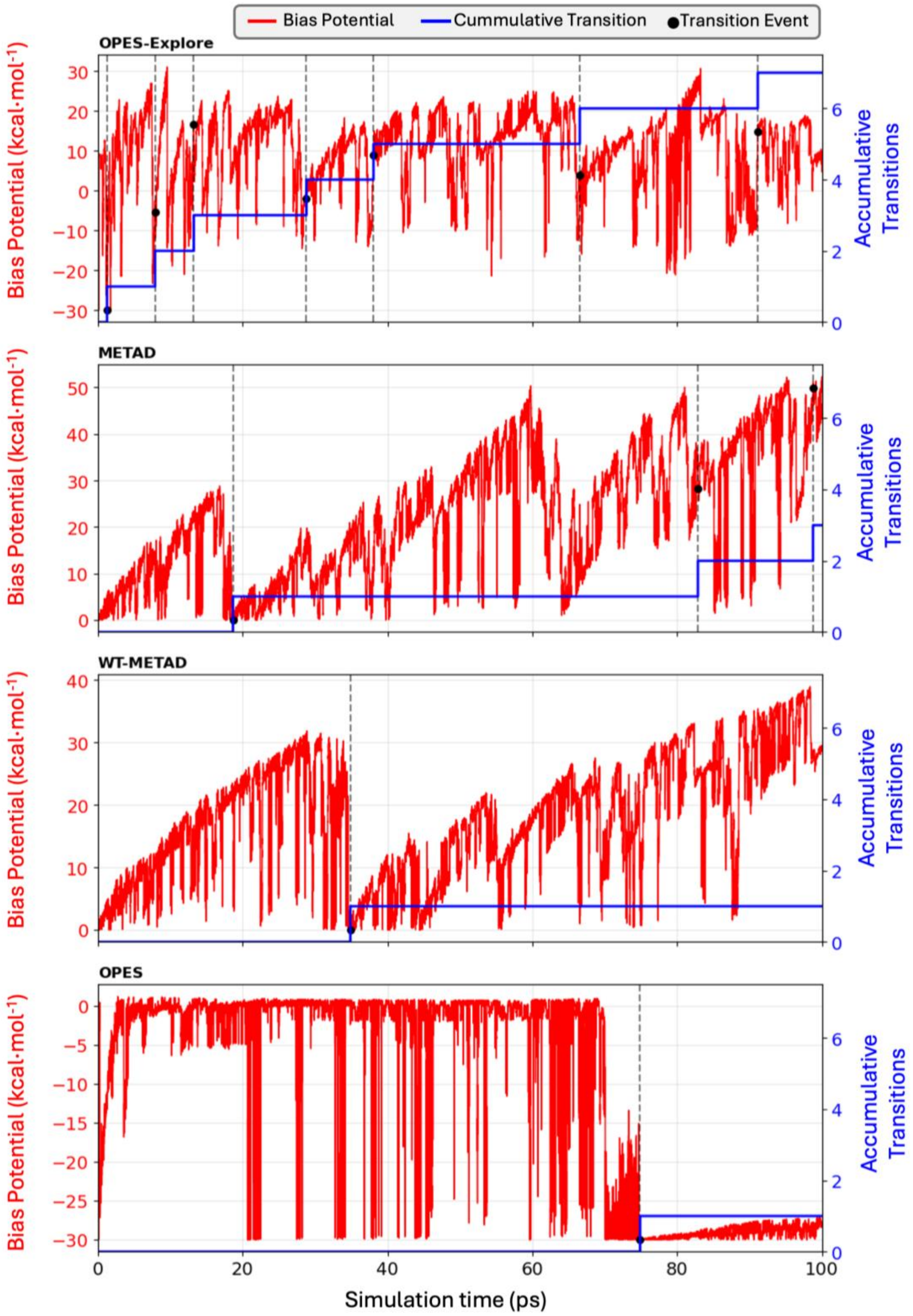

Figure S5. Evolution of the bias potential and cumulative number of transitions for each enhanced sampling method in simulations of the PL7A system. The method employed in each simulation is indicated in bold at the top-left corner of each panel. The x-axis represents the simulation time in picoseconds (ps). The left y-axis shows the external bias potential (red line) in kcal·mol^{-1}, while the right y-axis displays the cumulative number of transitions observed during the simulation (blue line). Black circles and grey dashed lines highlight the precise moments at which transitions occur. A transition is considered achieved when the system visits one metastable state immediately after escaping from the other, passing through the transition state region.

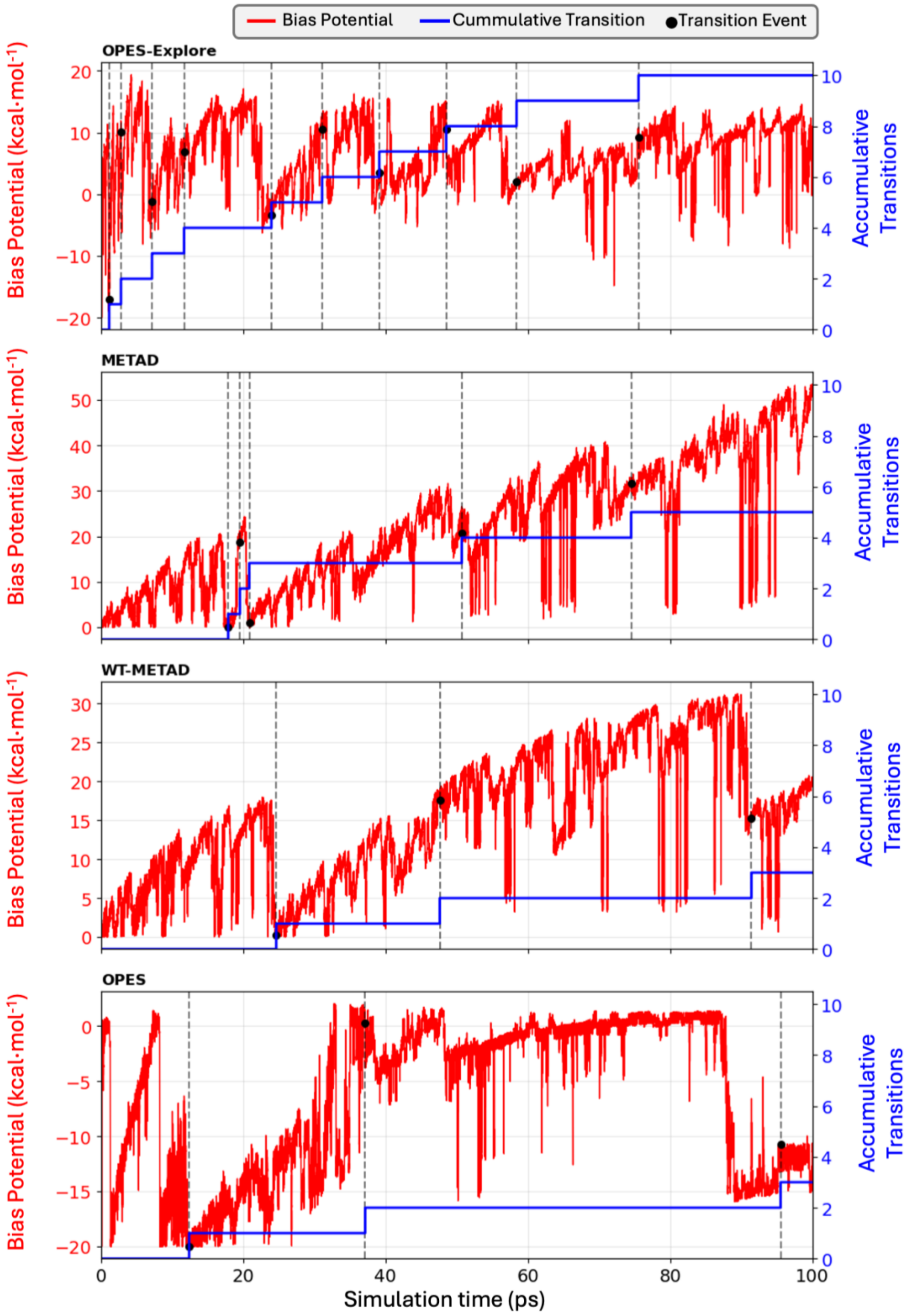

Figure S6. Evolution of the bias potential and cumulative number of transitions for each enhanced sampling method in simulations of the OGA system. The method employed in each simulation is indicated in bold at the top-left corner of each panel. The x-axis represents the simulation time in picoseconds (ps). The left y-axis shows the external bias potential (red line) in kcal·mol^{-1}, while the right y-axis displays the cumulative number of transitions observed during the simulation (blue line). Black circles and grey dashed lines highlight the precise moments at which transitions occur. A transition is considered achieved when the system visits one metastable state immediately after escaping from the other, passing through the transition state region.

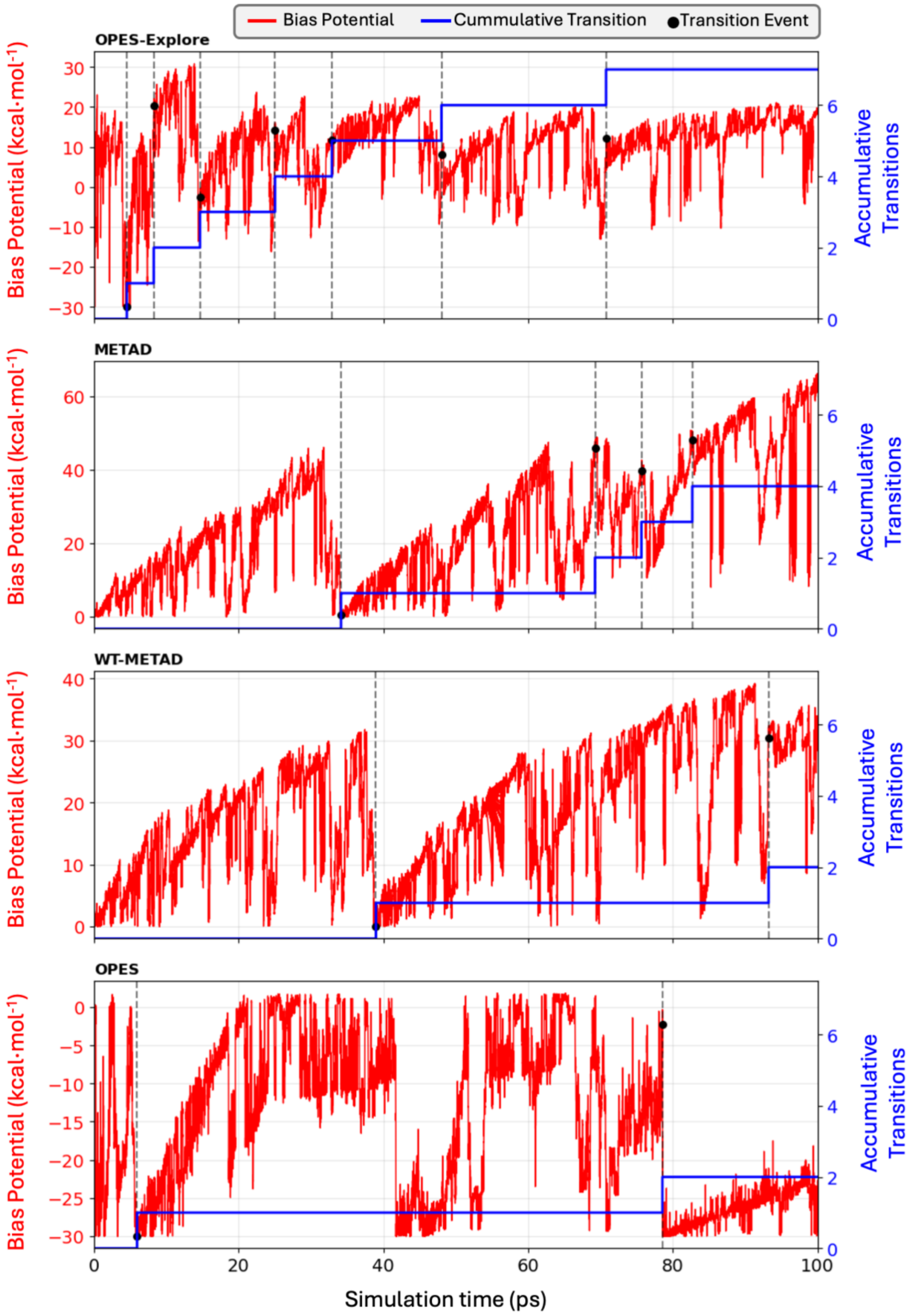

Figure S7. Evolution of the bias potential and cumulative number of transitions for each enhanced sampling method in simulations of the MPro system. The method employed in each simulation is indicated in bold at the top-left corner of each panel. The x-axis represents the simulation time in picoseconds (ps). The left y-axis shows the external bias potential (red line) in kcal·mol^{-1}, while the right y-axis displays the cumulative number of transitions observed during the simulation (blue line). Black circles and grey dashed lines highlight the precise moments at which transitions occur. A transition is considered achieved when the system visits one metastable state immediately after escaping from the other, passing through the transition state region.

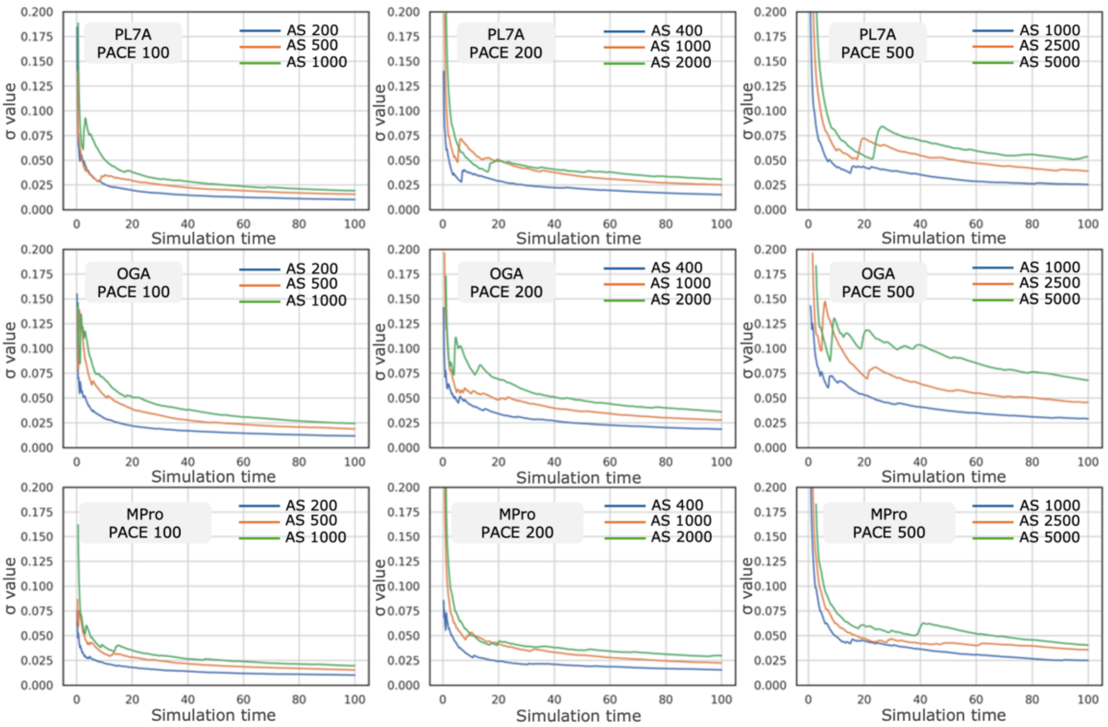

Figure S8. Evolution of sigma decay in the PathCV-OPES$_E$ simulations for different systems. The evolution of sigma values for the deposited kernels is shown for PL7A (top row), OGA (middle row), and MPro (bottom row). The y-axis represents sigma values, while the x-axis corresponds to the simulation time (in ps). Each plot is labelled with the PACE value used in the simulation. Three lines are displayed per plot, corresponding to the adaptive sigma (AS) settings: twice (blue), three times (orange), and five times (green) the PACE value.

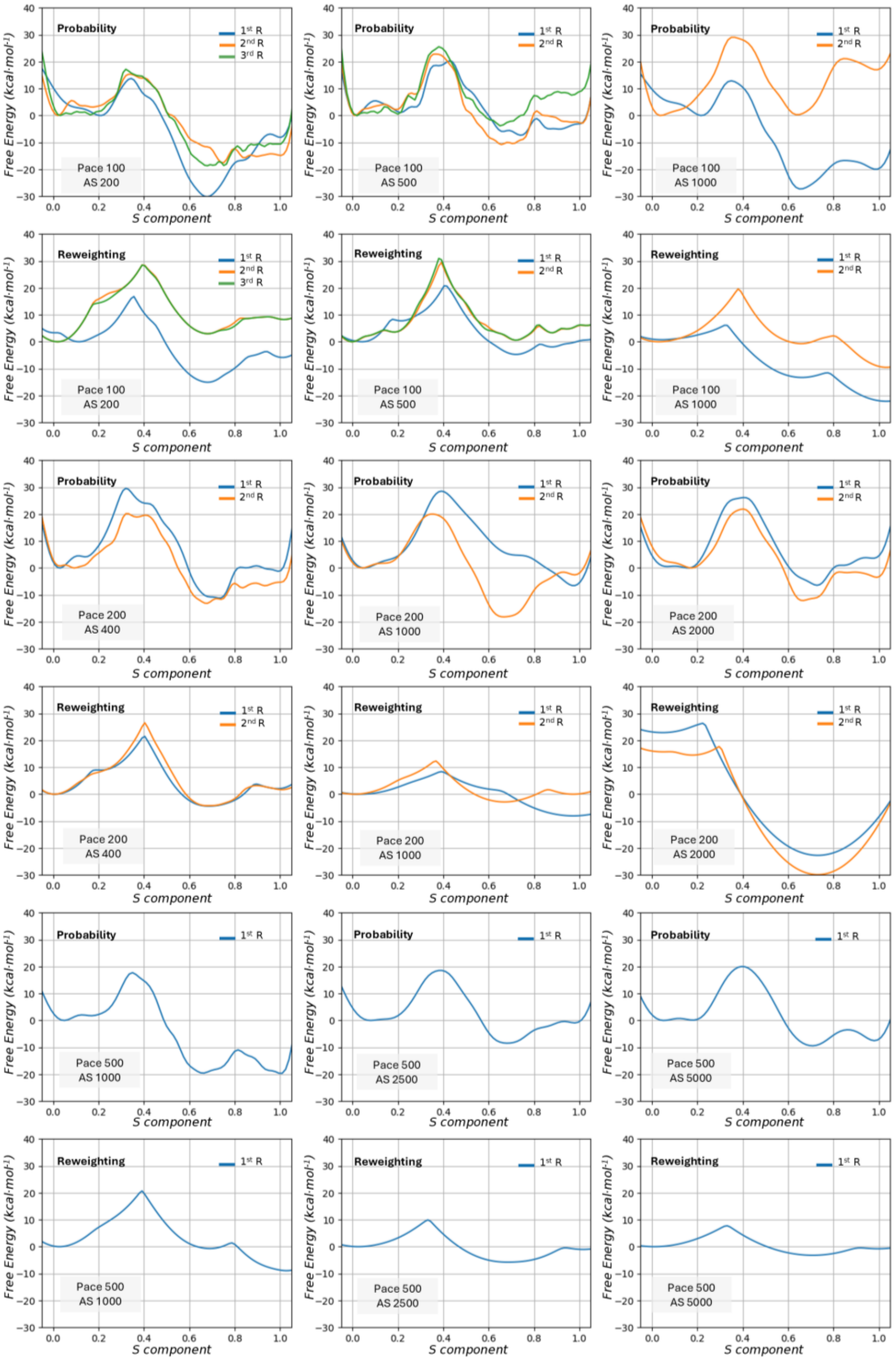

Figure S9. Free energy landscape computed after recrossing events for the PL7A system. The y-axis represents the computed free energy (in kcal·mol^{-1}), while the x-axis corresponds to the progression along the PathCV (the collective variable used in the simulation). Each panel is labeled with the method used for free energy computation (Probability Assessment or Reweighting) and the $OPES_E$ parameter settings applied in the simulations. The different lines in each panel represent the energetic profiles computed following the specified recrossing events.

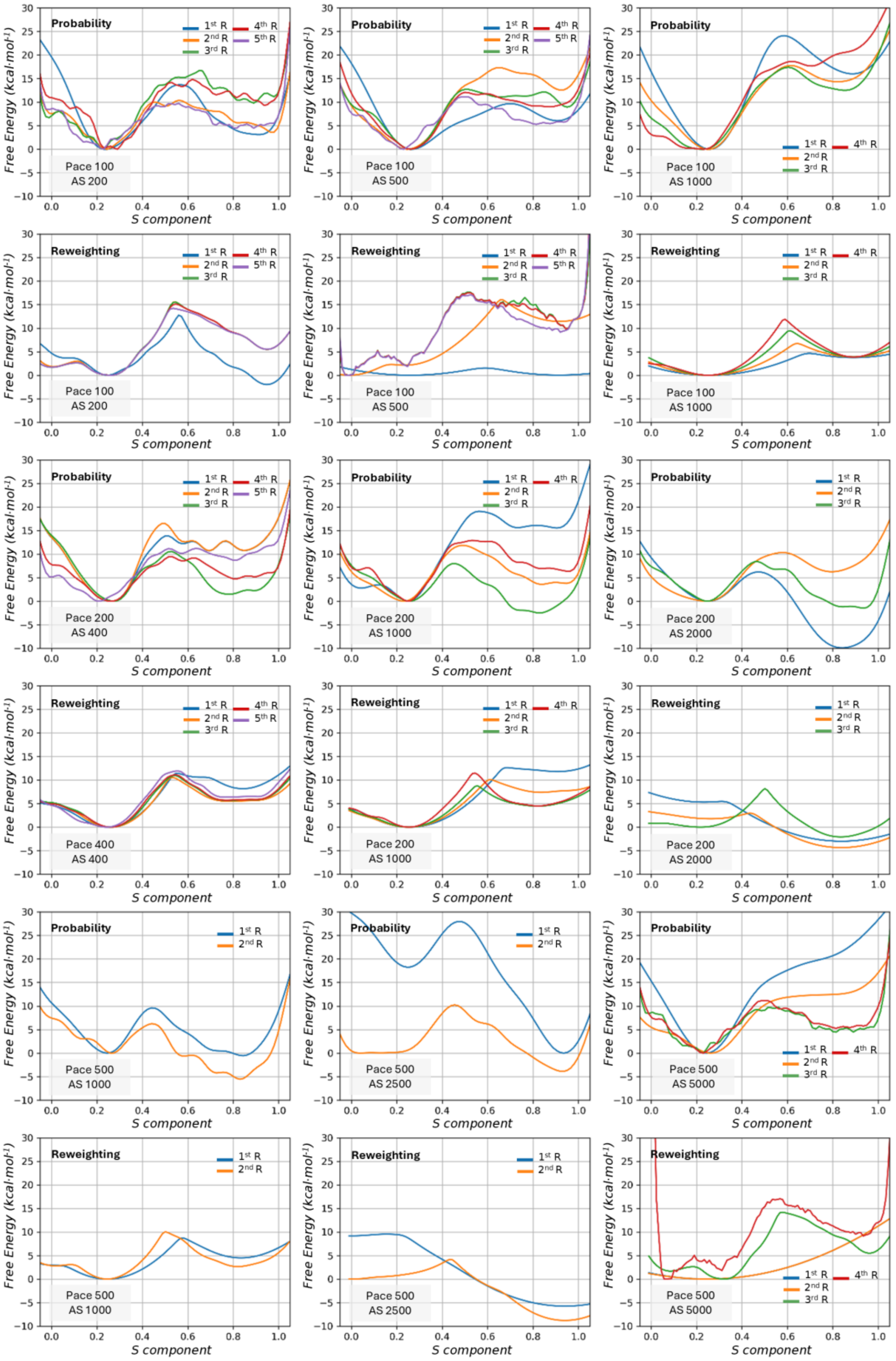

Figure S10. **Free energy landscape computed after recrossing events for the OGA system.** The y-axis represents the computed free energy (in kcal·mol^{-1}), while the x-axis corresponds to the progression along the PathCV (the collective variable used in the simulation). Each panel is labeled with the method used for free energy computation (Probability Assessment or Reweighting) and the OPES$_E$ parameter settings applied in the simulations. The different lines in each panel represent the energetic profiles computed following the specified recrossing events.

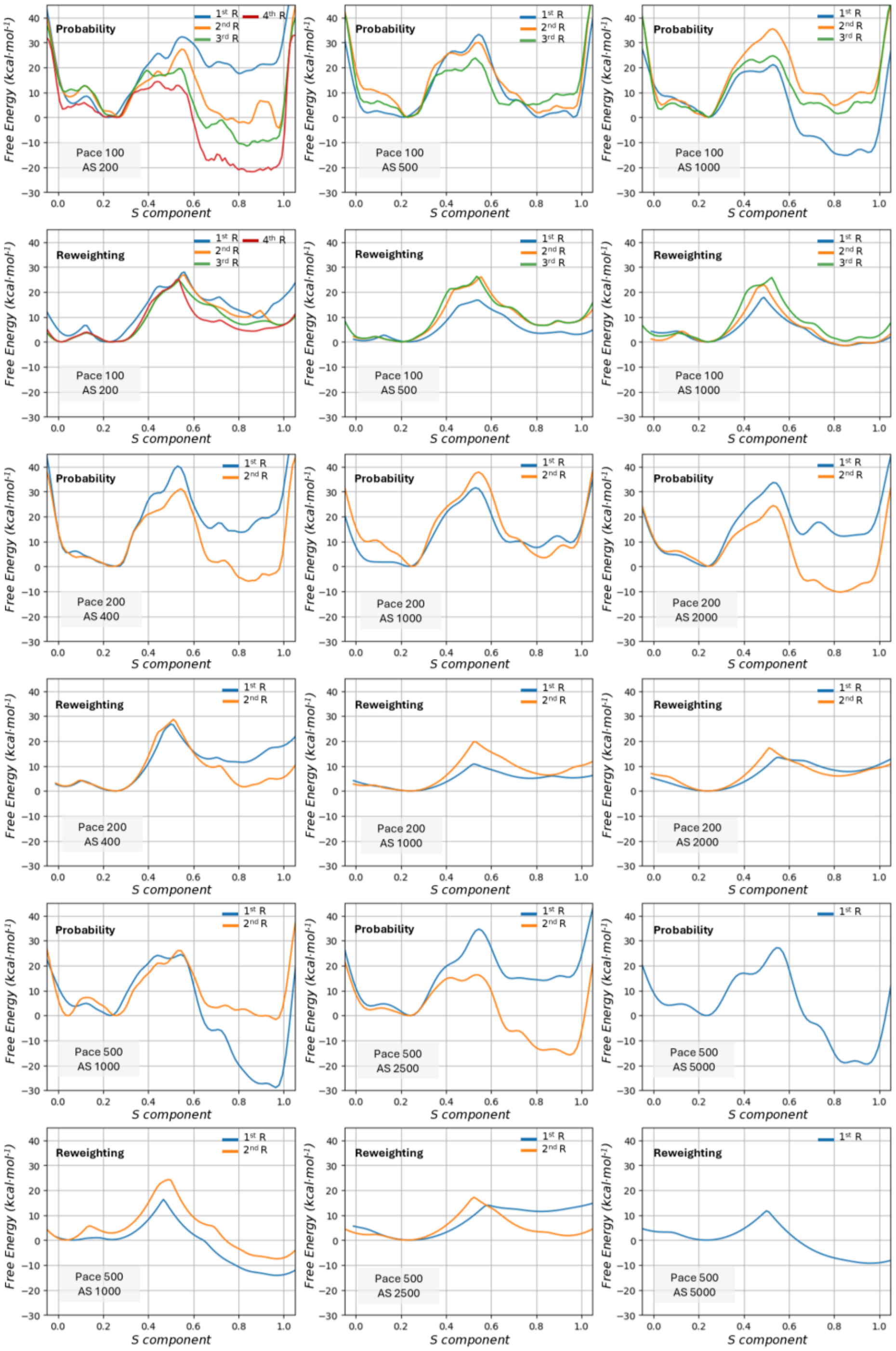

Figure S11. **Free energy landscape computed after recrossing events for the MPro system.** The y-axis represents the computed free energy (in kcal·mol^{-1}), while the x-axis corresponds to the progression along the PathCV (the collective variable used in the simulation). Each panel is labeled with the method used for free energy computation (Probability Assessment or Reweighting) and the OPES$_E$ parameter settings applied in the simulations. The different lines in each panel represent the energetic profiles computed following the specified recrossing events.

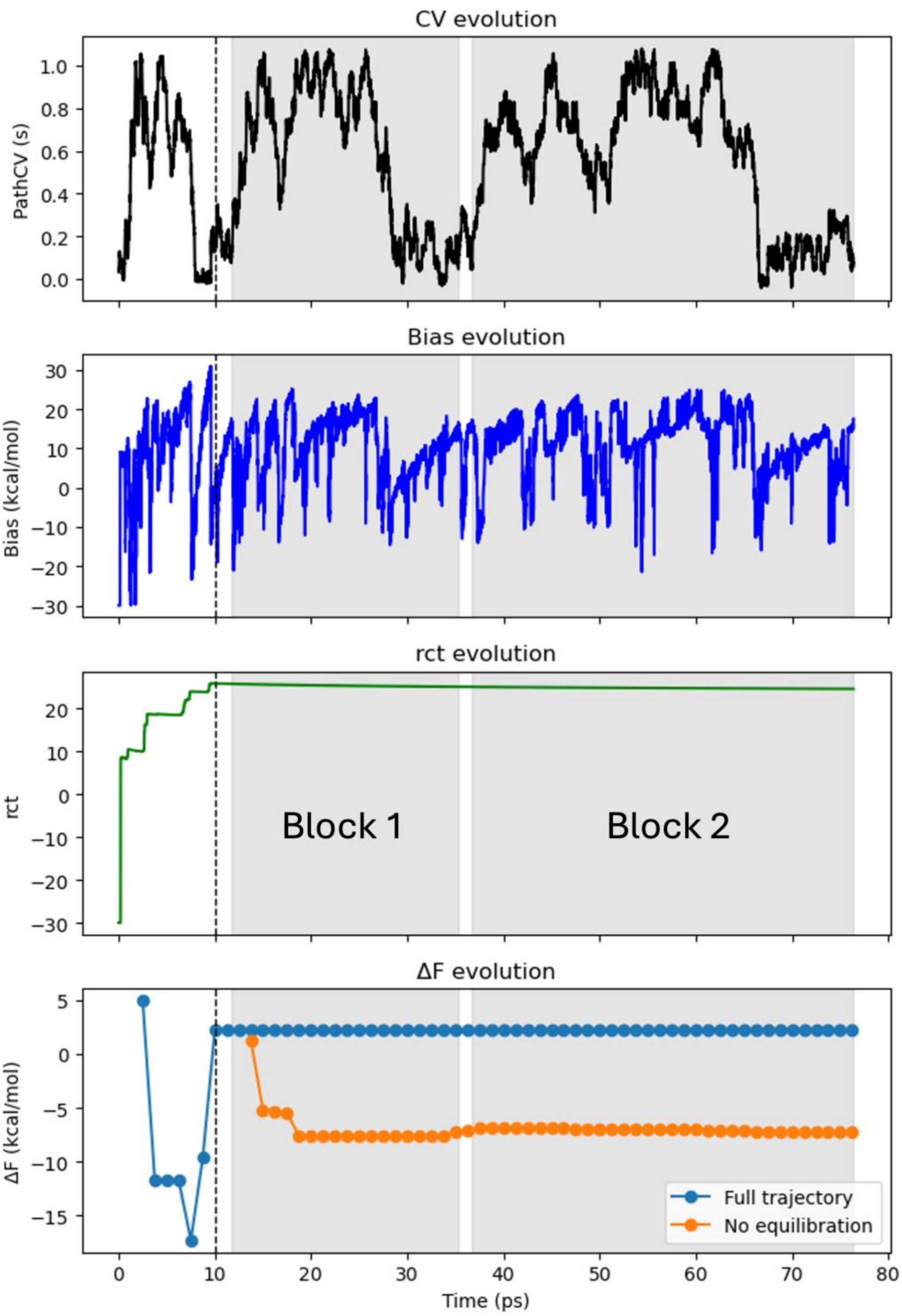

Figure S12. Illustration of the block-selection strategy and free energy convergence for the PL7A system. From top to bottom: evolution of the PathCV progress coordinate (*s*), bias potential, reweighting factor (*rct*), and free energy difference (ΔF) between reactant and product states. The vertical dashed line marks the end of the initial equilibration phase, defined by the stabilization of the *rct* parameter. Shaded regions indicate the trajectory segments used for analysis (Block 1 and Block 2). The ΔF evolution shows a strong dependence on the initial bias buildup, with large fluctuations at early times, while stable values are recovered when considering only post-equilibration blocks. Blue and orange curves correspond to ΔF computed from the full trajectory and from the post-equilibration region, respectively.

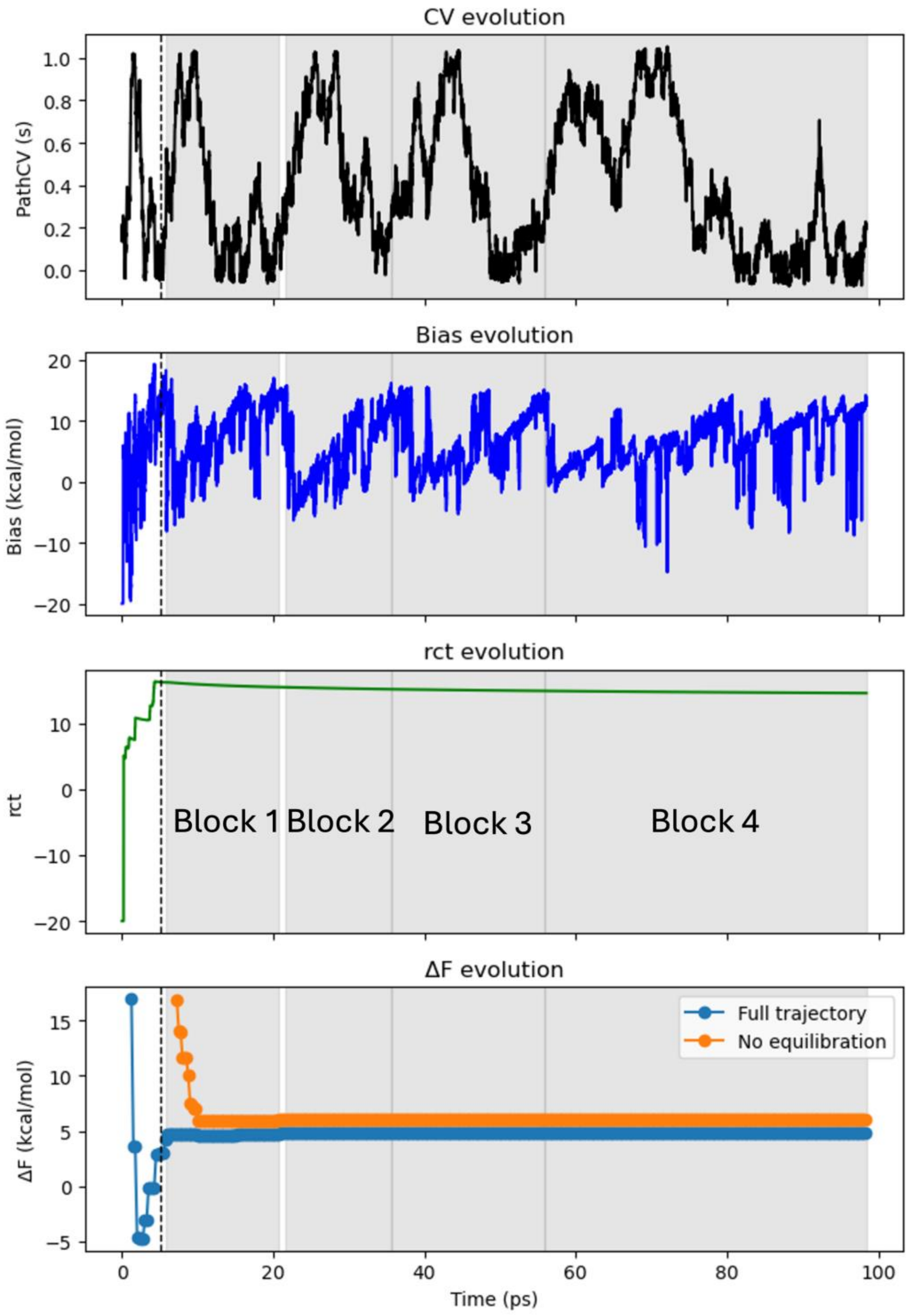

Figure S13. Illustration of the block-selection strategy and free energy convergence for the OGA system. From top to bottom: evolution of the PathCV progress coordinate (*s*), bias potential, reweighting factor (*rct*), and free energy difference (ΔF) between reactant and product states. The vertical dashed line marks the end of the initial equilibration phase, defined by the stabilization of the *rct* parameter. Shaded regions indicate the trajectory segments used for analysis (Block 1, Block 2, Block 3 and Block 4). The ΔF evolution shows a strong dependence on the initial bias buildup, with large fluctuations at early times, while stable values are recovered when considering only post-equilibration blocks. Blue and orange curves correspond to ΔF computed from the full trajectory and from the post-equilibration region, respectively.

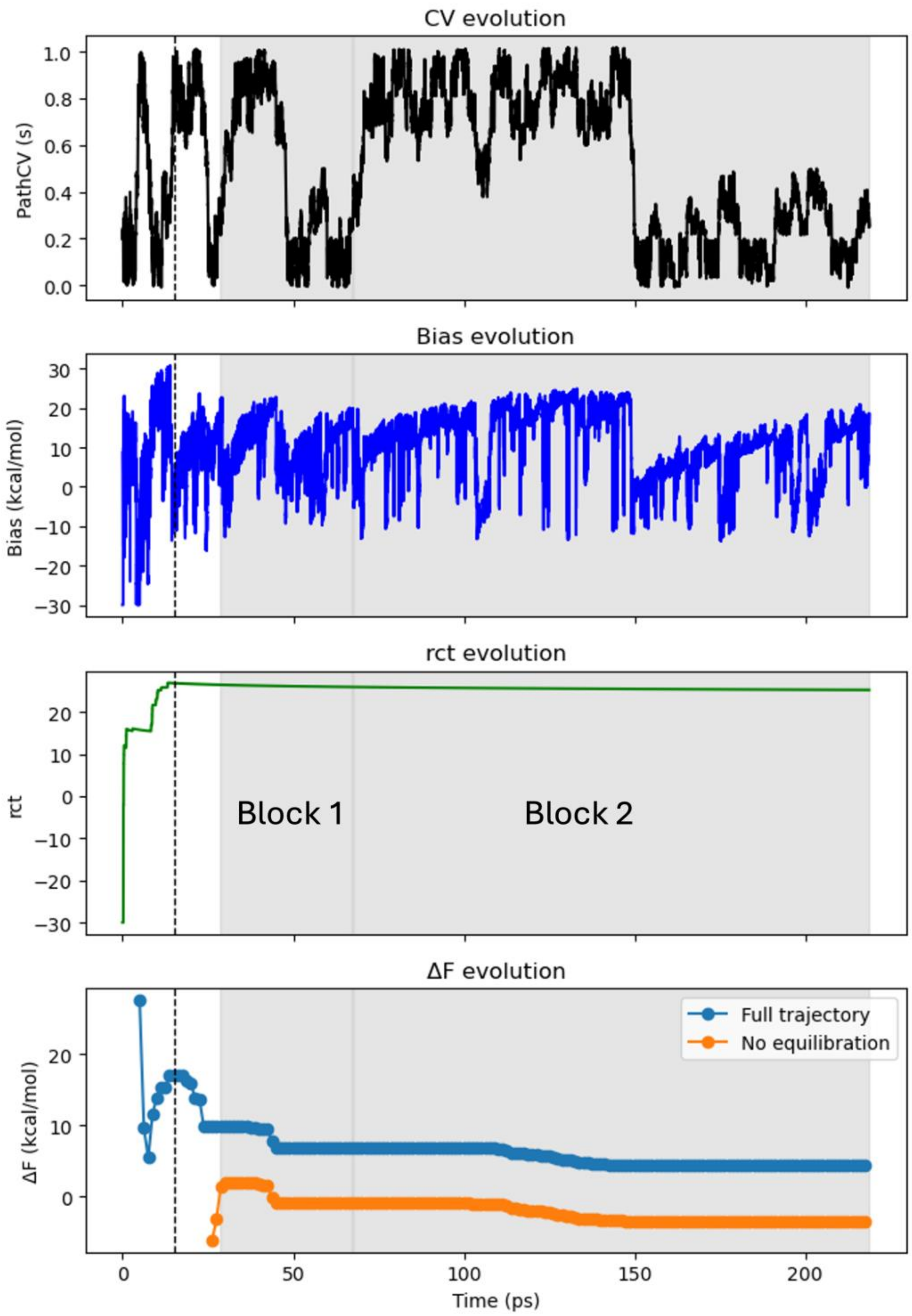

Figure S14. Illustration of the block-selection strategy and free energy convergence for the MPro system. From top to bottom: evolution of the PathCV progress coordinate (*s*), bias potential, reweighting factor (*rct*), and free energy difference (ΔF) between reactant and product states. The vertical dashed line marks the end of the initial equilibration phase, defined by the stabilization of the *rct* parameter. Shaded regions indicate the trajectory segments used for analysis (Block 1 and Block 2). The ΔF evolution shows a strong dependence on the initial bias buildup, with large fluctuations at early times, while stable values are recovered when considering only post-equilibration blocks. Blue and orange curves correspond to ΔF computed from the full trajectory and from the post-equilibration region, respectively.

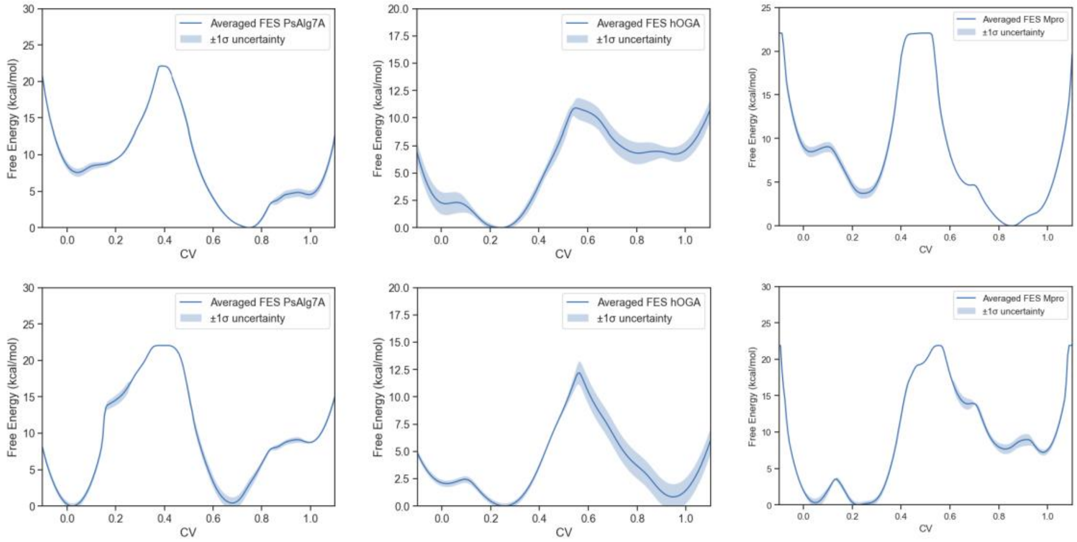

Figure S15. FELs reconstructed from block-selected trajectories. Top: FELs computed after discarding pre-transition frames corresponding to the bias-stabilization phase (prior to *rct* plateau). Bottom: FELs computed from the full trajectory, including the initial stabilization region.

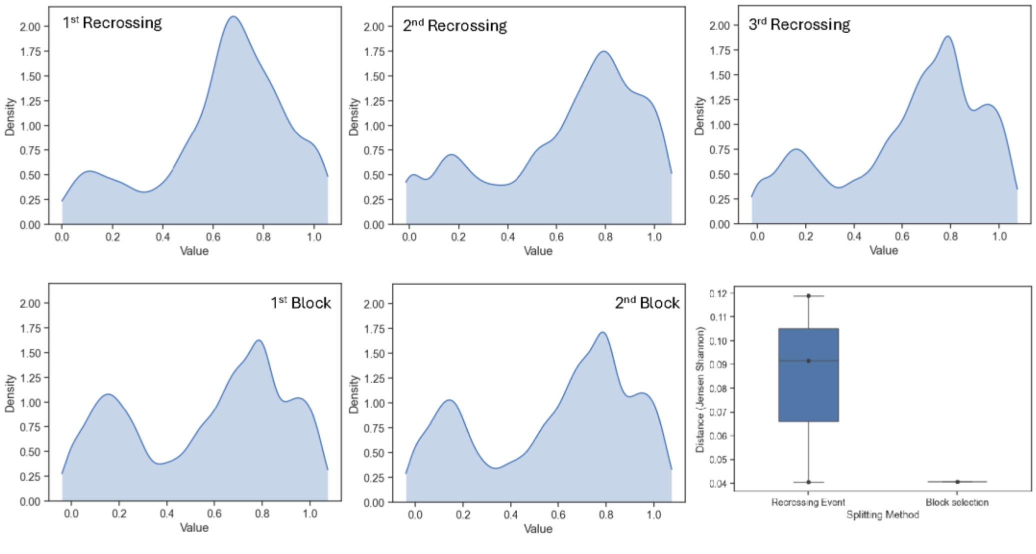

Figure S16. Probability density analysis for the PL7A system using different splitting methods. Top: densities extracted from sampling after a recrossing event. Bottom: densities obtained from block-selected trajectories, with Jensen–Shannon distances computed to quantify divergence between the resulting distributions.

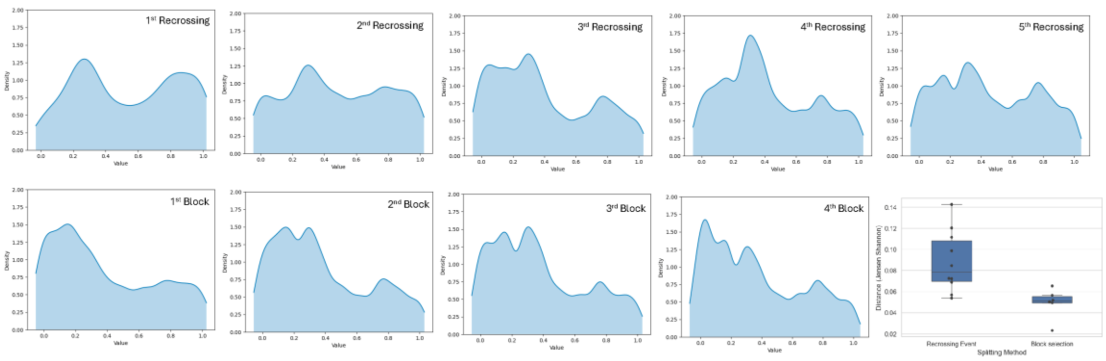

Figure S17. Probability density analysis for the OGA system using different splitting methods. Top: densities extracted from sampling after a recrossing event. Bottom: densities obtained from block-selected trajectories, with Jensen–Shannon distances computed to quantify divergence between the resulting distributions.

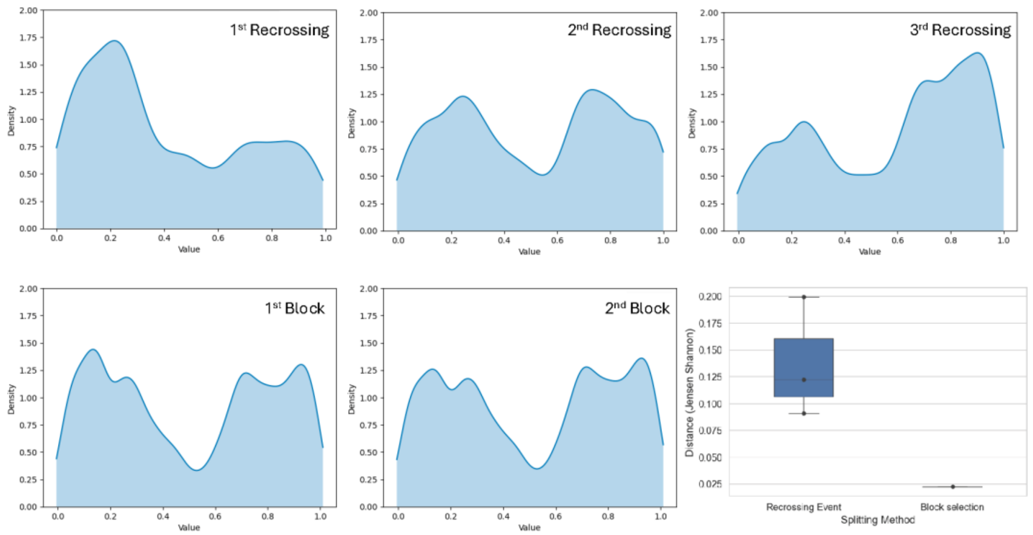

Figure S18. Probability density analysis for the MPro system using different splitting methods. Top: densities extracted from sampling after a recrossing event. Bottom: densities obtained from block-selected trajectories, with Jensen–Shannon distances computed to quantify divergence between the resulting distributions.

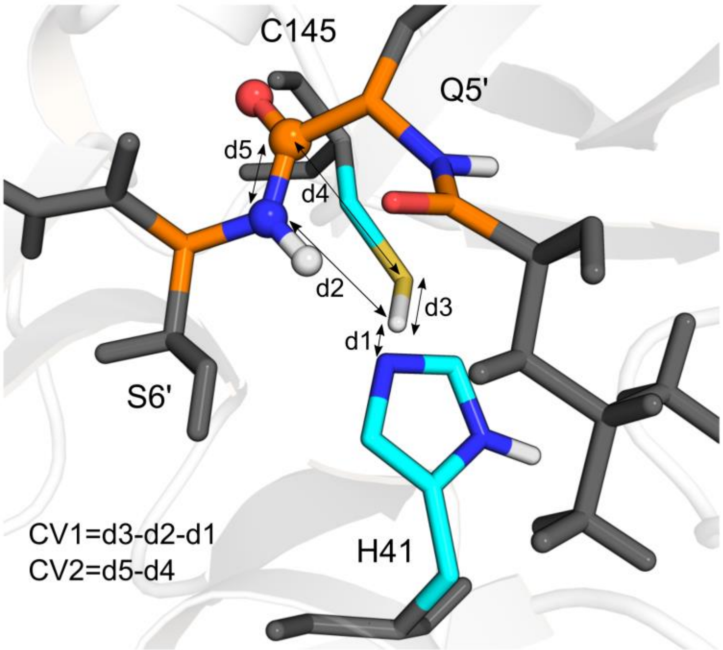

Figure S19. The illustration of the QM region used in the QM/MM simulations of MPro. The atoms in color are described by QM method (non-polar hydrogen atoms are hidden for clarity), while all the other atoms in grey are in the MM region described by force field. The distances used to define collective variables are labelled.

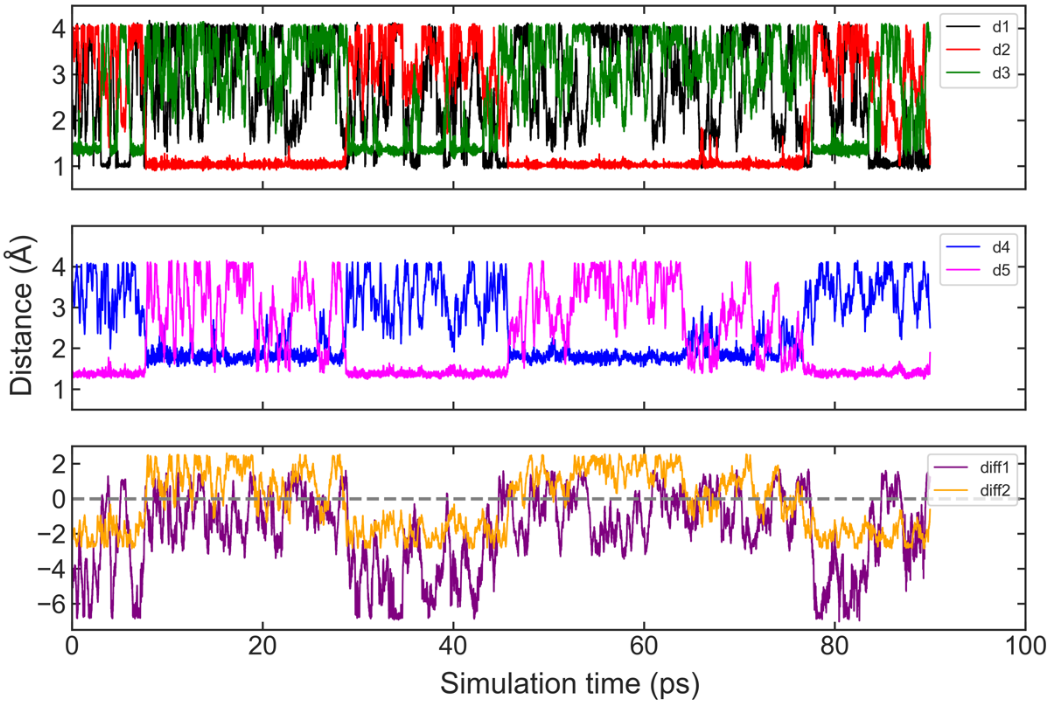

Figure S20. Time evolution of relevant distances during the QM/MM OPES-explore simulations. The two distance differences diff1 and diff2 were used as collective variables in this simulation. Details about the distances (d1 to d5, diff1 and diff2) are illustrated in **Figure S19**.

Table S1. Gaussian distribution parameters according to the s-component values used for defining the MC and P states in the block extraction. Mean values with their respective standard deviations are reported for each system, extracted from unbiased NVT simulations of the corresponding states.

Basin	Gaussian Parameter	PL7A	OGA	MPro
MC	Mean ± sigma	0.06±0.03	0.15±0.04	0.23±0.02
P	Mean ± sigma	0.68±0.04	0.87±0.06	0.83±0.03

Table S2. Summary of the simulations performed, with the different systems highlighted. PACE denotes the number of MD steps between kernel depositions. Adaptive Sigma (AS) indicates the number of MD steps at which the sigma of newly added kernels is recalculated, expressed in units of PACE. Time values are reported in picoseconds. The number of recrossings indicates the number of double transitions through the transition state that return to the same state from which the system started.

System	PACE	AS: times of PACE	Time	Number of recrossings
**PL7A**	100	2	106.3	3
		5	162.08	3
		10	180.50	2
	200	2	133.15	3
		5	131.79	2
		10	137.17	3
	500	2	109.14	1
		5	107.29	1
		10	99.89	1
**OGA**	100	2	100.62	7
		5	101.42	5
		10	100.83	4
	200	2	100.17	5
		5	121.69	4
		10	116.64	3
	500	2	101.02	3
		5	101.19	2
		10	100.77	5
**MPro**	100	2	218.75	4
		5	208.99	3
		10	225.99	3
	200	2	129.99	2
		5	112.47	2
		10	119.99	2
	500	2	185.00	2
		5	176.00	2
		10	185.00	1

Table S3. Information extracted from FELs computed after recrossing events in simulations of PL7A. For the different combinations of PACE and AS values, the activation barrier ($\Delta G^{\ddagger}$), the free energy difference between the MC and P states (ΔG), and the TS location (highest-energy point) along the ***s*** value coordinate are shown. These values were obtained from FELs reconstructed after recrossing events using both probability assessment and reweighting methods, as described in the manuscript. Energetic values are reported in kcal·mol^{-1}, and the TS location is given as a unidimensional ***s*** value ranging from 0 to 1.

PACE	AS	Recross	$\Delta G^{\ddagger}$ Probability	$\Delta G^{\ddagger}$ Reweighting	TS Probability	TS Reweighting	ΔG Probability	ΔG Reweighting
100	200	1	13.7	16.8	0.35	0.36	-30	-15.0
		2	15.47	28.4	0.34	0.39	-17.23	2.9
		3	17.08	28.4	0.32	0.40	-18.5	2.9
	500	1	20.5	20.8	0.42	0.4	-7.1	-4.71
		2	23.0	29.4	0.36	0.39	-11.0	0.6
		3	25.5	30.9	0.38	0.38	-3.8	0.6
	1000	1	12.9	5.4	0.35	0.32	-27.3	-22.83
		2	30.0	19.65	0.35	0.38	0.3	-9.5
200	400	1	29.5	21.5	40.6	0.41	-11.0	-4.33
		2	20.1	26.6	33.0	0.40	-12.9	-4.17
	1000	1	28.6	8.4	0.39	0.39	-6.33	-8.0
		2	20.0	12.4	0.36	0.37	-18.0	-2.8
	2000	1	26.1	3.45	0.41	0.22	-6.30	-45.6
		2	22.0	3.16	0.40	0.30	-12.0	-44.4
500	1000	1	17.6	20.7	0.35	0.39	-19.4	-8.8
	2500	1	18.5	10.1	0.39	0.33	-8.4	-5.6
	5000	1	20	7.75	0.39	0.33	-9.2	-3.2

Table S4. Information extracted from FELs computed after recrossing events in simulations of OGA. For the different combinations of PACE and AS values, the activation barrier ($\Delta G^{\ddagger}$), the free energy difference between the MC and P states (ΔG), and the TS location (highest-energy point) along the ***s*** value coordinate are shown. These values were obtained from FELs reconstructed after recrossing events using both probability assessment and reweighting methods, as described in the manuscript. Energetic values are reported in kcal·mol^{-1}, and the TS location is given as a unidimensional ***s*** value ranging from 0 to 1.

PACE	AS	Recross	$\Delta G^{\ddagger}$ Probability	$\Delta G^{\ddagger}$ Reweighting	TS Probability	TS Reweighting	ΔG Probability	ΔG Reweighting
100	2	1	13.7	12.7	0.56	0.56	3.1	-2.0
		2	10.4	15.5	0.56	0.55	3.6	5.5
		3	16.7	15.5	0.65	0.55	9.8	5.5
		4	14.8	15.1	0.62	0.55	9.3	5.5
		5	9.7	14.2	0.56	0.56	4.5	5.5
	5	1	9.7	1.5	0.70	0.58	6.1	0.0
		2	17.3	16.0	0.65	0.66	12.6	11.4
		3	12.7	17.7	0.50	0.51	9.0	9.9
		4	12.1	17.5	0.50	0.52	9.0	9.9
		5	11.1	17.1	0.50	0.52	5.3	9.1
	10	1	24.1	4.6	0.59	0.70	16.0	3.7
		2	17.8	6.7	0.62	0.64	14.3	3.9
		3	17.4	9.5	0.61	0.61	12.5	3.9
		4	18.7	11.9	0.62	0.59	17.7	3.9
200	2	1	13.9	11.7	0.51	0.56	10.7	8.5
		2	16.5	11.4	0.50	0.55	10.8	8.2
		3	10.5	10.5	0.53	0.55	1.5	5.5
		4	9.5	11.0	0.53	0.55	4.7	5.5
		5	11.2	11.1	0.52	0.55	8.7	5.7
	5	1	19.1	12.6	0.58	0.69	15.5	11.8
		2	11.9	10.1	0.49	0.61	3.6	7.4
		3	8.0	8.8	0.46	0.56	-2.5	4.5
		4	12.9	11.5	0.53	0.55	6.3	4.5
	10	1	6.2	0.1	0.47	0.32	-9.9	-8.3
		2	10.3	1.2	0.58	0.43	6.3	-6.1
		3	8.4	8.2	0.47	0.50	-1.5	-2.1
500	2	1	9.6	8.7	0.44	0.57	-0.6	4.5
		2	6.2	10.0	0.44	0.50	-5.5	2.6
	5	1	9.7	0.4	0.47	0.15	-18.2	-14.9
		2	10.2	4.2	0.46	0.43	-3.9	-8.8
	10	1	–	–	–	–	–	–
		2	–	–	–	–	–	–
		3	12.6	–	0.58	–	9.8	–
		4	10.5	–	0.52	–	3.1	–

Table S5. Information extracted from FELs computed after recrossing events in simulations of MPro. For the different combinations of PACE and AS values, the activation barrier ($\Delta G^{\ddagger}$), the free energy difference between the MC and P states (ΔG), and the TS location (highest-energy point) along the ***s*** value coordinate are shown. These values were obtained from FELs reconstructed after recrossing events using both probability assessment and reweighting methods, as described in the manuscript. Energetic values are reported in kcal·mol^{-1}, and the TS location is given as a unidimensional ***s*** value ranging from 0 to 1.

PACE	AS	Recross	$\Delta G^{\ddagger}$ Probability	$\Delta G^{\ddagger}$ Reweighting	TS Probability	TS Reweighting	ΔG Probability	ΔG Reweighting
100	2	1	32.25	28.0	0.55	0.56	17.6	9.64
		2	27.2	30.0	0.55	0.56	-4.4	6.75
		3	19.5	26.3	0.54	0.54	-11.5	6.8
		4	14.4	22.7	0.44	0.52	-21.8	4.28
	5	1	33.3	16.6	0.55	0.55	0	3
		2	30	26	0.55	0.56	1.8	6.6
		3	23.8	26.3	0.53	0.54	5.0	6.6
	10	1	21.1	17.9	0.53	0.49	-15.3	-1.5
		2	35.5	11.9	0.53	0.49	4.8	-1.5
		3	24.7	25.8	0.53	0.52	1.5	0.4
200	2	1	40.2	26.8	0.53	0.50	13.7	11.5
		2	31.1	28.7	0.55	0.51	-5.9	1.7
	5	1	31.0	10.8	0.53	0.52	7.2	5.1
		2	37.5	19.7	0.54	0.52	3.2	6.3
	10	1	33.5	13.4	0.54	0.56	12	7.7
		2	24.5	17.3	0.53	0.52	-9.9	5.9
500	2	1	24.5	16.3	0.55	0.47	-28.8	-14.1
		2	27.1	24.2	0.55	0.79	-0.05	-7.5
	5	1	34.7	14.0	0.54	0.58	14.1	15.5
		2	16.3	17.2	0.53	0.52	-15.8	1.8
	10	1	27.1	11.7	0.55	0.50	-19.5	-9.3

Table S6. Reference values for the activation barrier (ΔG‡) and the free energy difference between reactant and product states (ΔG) used in this analysis. These energetic values were obtained from prior computational studies and from experimental reaction rates using transition-state theory and the Eyring equation. All values are reported in kcal·mol^{-1}.

System	ΔG‡ (kcal·mol^{-1})	ΔG (kcal·mol^{-1})	References
PL7A	17.6	-10	18,19
OGA	16-18	5.4	20–23
MPro	17.8	<0	1,24

REFERENCES

(1) MacDonald, E. A.; Frey, G.; Namchuk, M. N.; Harrison, S. C.; Hinshaw, S. M.; Windsor, I. W. Recognition of Divergent Viral Substrates by the SARS-CoV-2 Main Protease. *ACS Infect Dis* **2021**, *7* (9), 2591–2595.

(2) Meng, E. C.; Goddard, T. D.; Pettersen, E. F.; Couch, G. S.; Pearson, Z. J.; Morris, J. H.; Ferrin, T. E. UCSF ChimeraX: Tools for Structure Building and Analysis. *Protein Science* **2023**, *32* (11).

(3) Maier, J. A.; Martinez, C.; Kasavajhala, K.; Wickstrom, L.; Hauser, K. E.; Simmerling, C. Ff14SB: Improving the Accuracy of Protein Side Chain and Backbone Parameters from Ff99SB. *J Chem Theory Comput* **2015**, *11* (8), 3696–3713.

(4) D.A. Case; H.M. Aktulga; K. Belfon; I.Y. Ben-Shalom; S.R. Brozell; et all. *Amber 2021*; San Francisco, 2021.

(5) Götz, A. W.; Williamson, M. J.; Xu, D.; Poole, D.; Le Grand, S.; Walker, R. C. Routine Microsecond Molecular Dynamics Simulations with AMBER on GPUs. 1. Generalized Born. *J Chem Theory Comput* **2012**, *8* (5), 1542–1555.

(6) Ryckaert, J.-P.; Ciccotti, G.; Berendsen, H. J. C. Numerical Integration of the Cartesian Equations of Motion of a System with Constraints: Molecular Dynamics of n-Alkanes. *J Comput Phys* **1977**, *23* (3), 327–341.

(7) Karplus, M. Molecular Dynamics Simulations of Biomolecules. *Acc Chem Res* **2002**, *35* (6), 321–323.

(8) Warshel, A.; Levitt, M. Theoretical Studies of Enzymic Reactions: Dielectric, Electrostatic and Steric Stabilization of the Carbonium Ion in the Reaction of Lysozyme. *J Mol Biol* **1976**, *103* (2), 227–249.

(9) Woo, T. K.; Cavallo, L.; Ziegler, T. Implementation of the IMOMM Methodology for Performing Combined QM/MM Molecular Dynamics Simulations and Frequency Calculations. *Theoretical Chemistry Accounts: Theory, Computation, and Modeling (Theoretica Chimica Acta)* **1998**, *100* (5–6), 307–313.

(10) Hutter, J.; Iannuzzi, M.; Schiffmann, F.; VandeVondele, J. Cp2k: Atomistic Simulations of Condensed Matter Systems. *WIREs Computational Molecular Science* **2014**, *4* (1), 15–25.

(11) The PLUMED consortium. Promoting Transparency and Reproducibility in Enhanced Molecular Simulations. *Nat Methods* **2019**, *16* (8), 670–673.

(12) Goedecker, S.; Teter, M.; Hutter, J. Separable Dual-Space Gaussian Pseudopotentials. *Phys Rev B* **1996**, *54* (3), 1703–1710.

(13) Liu, D. C.; Nocedal, J. On the Limited Memory BFGS Method for Large Scale Optimization. *Math Program* **1989**, *45* (1–3), 503–528.

(14) Bussi, G.; Donadio, D.; Parrinello, M. Canonical Sampling through Velocity Rescaling. *J Chem Phys* **2007**, *126* (1).

(15) Invernizzi, M.; Parrinello, M. Exploration vs Convergence Speed in Adaptive-Bias Enhanced Sampling. *J Chem Theory Comput* **2022**, *18* (6), 3988–3996.

(16) Díaz Leines, G.; Ensing, B. Path Finding on High-Dimensional Free Energy Landscapes. *Phys Rev Lett* **2012**, *109* (2), 020601.

(17) Pérez de Alba Ortíz, A.; Tiwari, A.; Puthenkalathil, R. C.; Ensing, B. Advances in Enhanced Sampling along Adaptive Paths of Collective Variables. *J Chem Phys* **2018**, *149* (7).

(18) Pilgaard, B.; Vuillemin, M.; Holck, J.; Wilkens, C.; Meyer, A. S. Specificities and Synergistic Actions of Novel PL8 and PL7 Alginate Lyases from the Marine Fungus Paradendryphiella Salina. *Journal of Fungi* **2021**, *7* (2), 80.

(19) Rivas-Fernández, J. P.; Vuillemin, M.; Pilgaard, B.; Klau, L. J.; Fredslund, F.; Lund-Hanssen, C.; Welner, D. H.; Meyer, A. S.; Morth, J. P.; Meilleur, F.; Aachmann, F. L.; Rovira, C.; Wilkens, C. Unraveling the Molecular Mechanism of Polysaccharide Lyases for Efficient Alginate Degradation. *Nat Commun* **2025**, *16* (1), 2670.

(20) Kiss, M.; Szabó, E.; Bocska, B.; Sinh, L. T.; Fernandes, C. P.; Timári, I.; Hayes, J. M.; Somsák, L.; Barna, T. Nanomolar Inhibition of Human OGA by 2-Acetamido-2-Deoxy-d-Glucono-1,5-Lactone Semicarbazone Derivatives. *Eur J Med Chem* **2021**, *223*, 113649.

(21) Calvelo, M.; Males, A.; Alteen, M. G.; Willems, L. I.; Vocadlo, D. J.; Davies, G. J.; Rovira, C. Human O-GlcNAcase Uses a Preactivated Boat-Skew Substrate Conformation for Catalysis. Evidence from X-Ray Crystallography and QM/MM Metadynamics. *ACS Catal* **2023**, *13* (20), 13672–13678.

(22) Macauley, M. S.; Whitworth, G. E.; Debowski, A. W.; Chin, D.; Vocadlo, D. J. O-GlcNAcase Uses Substrate-Assisted Catalysis. *Journal of Biological Chemistry* **2005**, *280* (27), 25313–25322. +

(23) Kim, E. J.; Kang, D. O.; Love, D. C.; Hanover, J. A. Enzymatic Characterization of O-GlcNAcase Isoforms Using a Fluorogenic GlcNAc Substrate. *Carbohydr Res* **2006**, *341* (8), 971–982.

(24) Ramos-Guzmán, C. A.; Ruiz-Pernía, J. J.; Tuñón, I. Unraveling the SARS-CoV-2 Main Protease Mechanism Using Multiscale Methods. *ACS Catal* **2020**, *10* (21), 12544–12554.
